# Extracellular matrix stiffness cues junctional remodeling for 3D tissue elongation

**DOI:** 10.1038/s41467-019-10874-x

**Published:** 2019-07-26

**Authors:** Dong-Yuan Chen, Justin Crest, Sebastian J. Streichan, David Bilder

**Affiliations:** 1 Department of Molecular and Cell Biology, University of California, Berkeley, Berkeley, CA 94720-3200 USA; 2 Department of Physics, University of California, Santa Barbara Santa, Barbara, CA 93106-9530 USA

**Keywords:** Cell adhesion, Cell division, Cell migration, Morphogenesis

## Abstract

Organs are sculpted by extracellular as well as cell-intrinsic forces, but how collective cell dynamics are orchestrated in response to environmental cues is poorly understood. Here we apply advanced image analysis to reveal extracellular matrix-responsive cell behaviors that drive elongation of the *Drosophila* follicle, a model system in which basement membrane stiffness instructs three-dimensional tissue morphogenesis. Through in toto morphometric analyses of wild type and round egg mutants, we find that neither changes in average cell shape nor oriented cell division are required for appropriate organ shape. Instead, a major element is the reorientation of elongated cells at the follicle anterior. Polarized reorientation is regulated by mechanical cues from the basement membrane, which are transduced by the Src tyrosine kinase to alter junctional E-cadherin trafficking. This mechanosensitive cellular behavior represents a conserved mechanism that can elongate edgeless tubular epithelia in a process distinct from those that elongate bounded, planar epithelia.

## Abstract (web summary)

The extracellular matrix can shape developing organs, but how external forces direct intercellular morphogenesis is unclear. Here, the authors use 3D imaging to show that elongation of the Drosophila egg chamber involves polarized cell reorientation signalled by changes in stiffness of the surrounding extracellular matrix.

## Introduction

A long-time goal of biology is to understand the full set of mechanisms that shape a functional organ. Many morphogenesis studies have focused on only a part of the organ, either by culturing dissected portions ex vivo, or by restricting in vivo imaging to an optically convenient region. Fundamental morphogenetic principles have emerged from classical experimental systems such as Keller explants of Xenopus embryos^1^, as well as contemporary examples such as the *Drosophila* germband^2^. However, in the former case, tissue is physically removed from its native environment, whereas in the latter only a portion of the tissue is imaged. Such approaches introduce artificial boundaries to the tissue, which limits evaluation of outside influences including tissue-wide mechanics. Only recently have comprehensive analyses of systems like the *Drosophila* notum and wing imaginal disc, zebrafish gastrula and avian embryo commenced^3^. Nevertheless, these tissues tend to be treated primarily as two-dimensional sheets, in contrast to the many in vivo organs that contain multiple tissue types organized in three dimensions (3D). Thus, there is a need to study true 3D organs with in toto approaches.

The *Drosophila* egg chamber, or follicle, provides an excellent model for this goal. Follicles have an architecture that is typical of a number of animal organs, with several components that associate to form a 3D acinar epithelium surrounding a lumen^4^. At the same time, the simplicity and highly regular development of the follicle lend themselves to comprehensive analyses. The follicle exhibits straightforward and symmetric geometry for much of its development, while its cells originate from only two stem cell populations and show limited differential fates^5^. Follicles can be genetically manipulated using the powerful *Drosophila* toolkit, and are well-suited for imaging either in fixed preparations or when cultured live ex vivo.

Development of the follicle involves several conserved morphogenetic behaviors including initial primordial assembly, epithelial diversification, and collective cell migration. A major focus for mechanistic studies has been follicle elongation, during which the initially spherical organ transforms into a more tube-like ellipsoid shape^5,6^. ~2-fold elongation is seen in ~40 h between follicle budding at stage 3 to the end of stage 8; eventually there is ~2.5-fold overall elongation when the egg is laid ~25 h later. This degree of elongation is similar to that in paradigmatic morphogenetic systems such as the amphibian neural plate and mesoderm, or the *Drosophila* germband. In the latter tissues, the main cellular behavior that drives elongation is convergent extension, as cells intercalate mediolaterally toward a specific landmark that is defined anatomically and/or molecularly. However, these tissues have defined borders, which create boundary conditions to instruct and orient cell behaviors. No such boundary is evident along the edgeless epithelium of the *Drosophila* follicle^7^, and the cellular changes that drive elongation of this acinar organ are not known.

We recently showed that mechanical heterogeneity patterned not within the cells of the follicle, but instead within its underlying basement membrane (BM), instructs organ shape^8^. Specifically, a gradient of matrix stiffness that is low at the poles and peaks in the organ center provides differential resistance to luminal expansion, leading to tissue elongation. Construction of this pattern relies in part on a collective migration of cells around the follicle equatorial axis, leading to global tissue rotation^9^. But how the cells of the epithelium respond to stiffness cues and engage in the dynamics that actually elongate the organ along the anterior-posterior (A–P) axis remains unexplored.

Here we identify an unexpected cell behavior that drives follicle elongation and demonstrate its control by a regulatory axis that responds to BM stiffness cues, thus connecting extracellular mechanical properties to intracellular signaling that drives intercellular morphogenesis in vivo.

## Results

### In toto morphometrics of *Drosophila* follicles

**Fig. 1:**
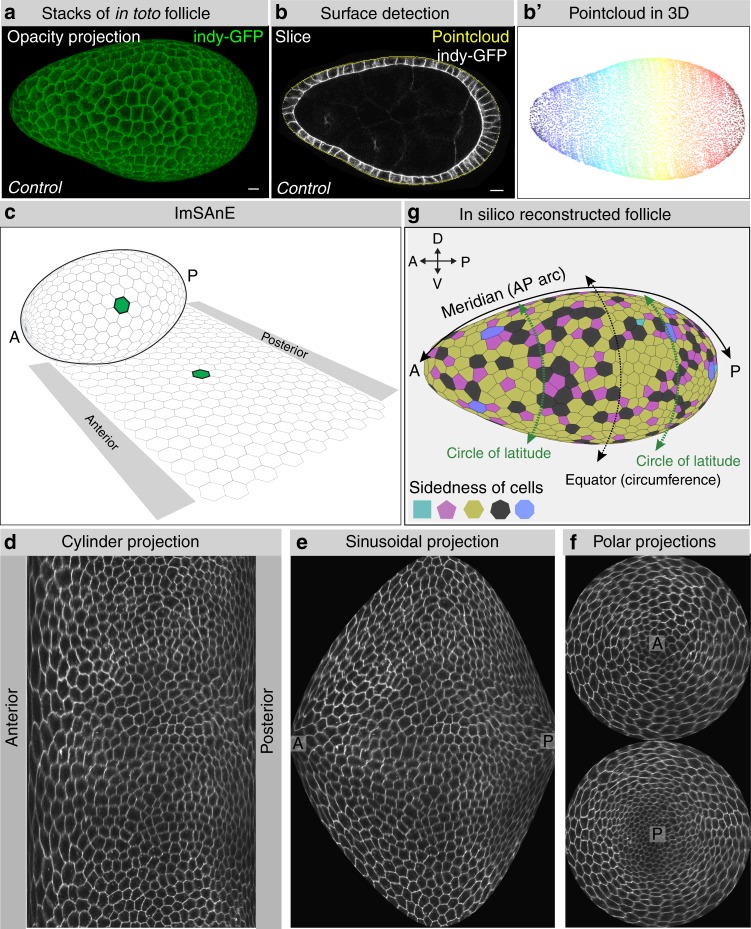
Workflow for in toto follicle morphometrics extraction. **a** Full 3D stacks of non-compressed Indy-GFP (green) expressing follicles are obtained with confocal microscopy (**b**), after which a 3D pointcloud (**b**′) is extracted from the detected surface (**b**). **c**–**f** ImSAnE then converts the pointcloud into various 2D projections of segmented cells. **g** Projections and segmentation veracity are confirmed by 3D reconstruction of the follicle (see also Supplementary Movie 1). This workflow enables analyses of poles as well as axes along the follicle epithelium, as defined in (**g**), meridians connecting anterior (A) and posterior (P) poles, and circles of latitudes including the equator (circumference). Scale bars, 10 μm

We established an imaging and computational platform to acquire morphometric data from follicles during their major elongation phase, from stage 4 to stage 8, prior to major asymmetries between the anterior and posterior hemispheres (see “Methods” section). Because morphometric measurements can be altered by mounting preparation (e.g. flattened on a slide and/or coverslip) and by artifacts of imaging plane (e.g. XY slices of cells that also have Z orientation in the epithelial plane), complete XYZ images of follicles within a depression slide were captured on a confocal microscope, and analyzed using advanced software including the recently described package ImSAnE^7,10^ (Fig. 1a–c). lmSAnE detects the surfaces of 3D objects and projects them onto 2D planes to facilitate quantitative analysis and visualization. Various projections, displaying slices of cells at their basal surface, can be selected that reflect either the size or orientation with respect to axes within the plane of the follicle epithelium (Fig. 1d–f; see Fig. 1g for definition of meridional and latitudinal axes). Despite the distortions intrinsic to all 2D projections, accurate morphometric data is preserved in the software. The veracity of ImSAnE projections was confirmed by the ability to reconstruct 3D follicles in silico (Fig. 1g and Supplementary Movie 1). Importantly, this approach allowed complete analysis of the epithelium, including both polar regions, which are seldom scrutinized due to their high curvature and their position perpendicular to the standard imaging plane.

**Fig. 2:**
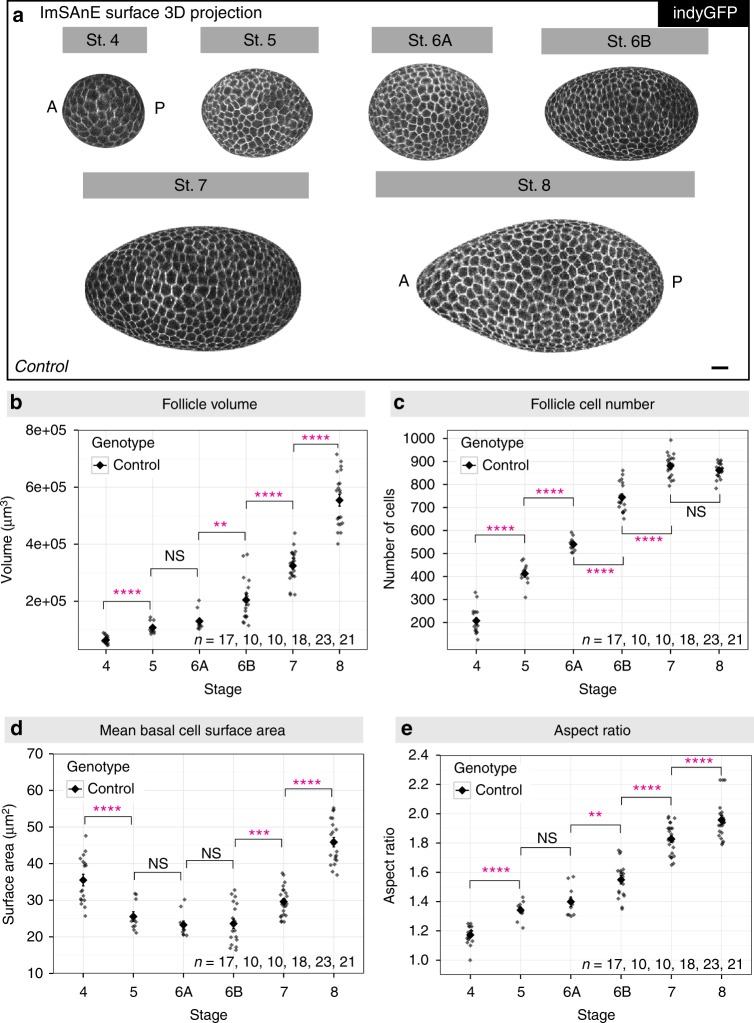
Tissue and cellular level morphometrics of wild-type follicles. **a** Representative ImSAnE surface 3D projection of Indy-GFP (gray) expressing follicles at stage 4–8. Note the increased elongation of stage 6B follicles as compared to stage 6 A. Scale bar, 10 μm. **b** Follicle volume increases ~exponentially during these stages, ~9-fold from stage 4–8. *P* values = 7.1e-05, 0.09, 0.0015, 4.6e-06, and 4.4e-11; two-sided Welch’s *t* test. **c** Follicle cell number increases ~linearly with stage, correlates with most morphological criteria, and can be used to define boundaries for each stage. Follicles at stage 7 have reached their maximum cell number. *P* values = 2.5e-09, 5.1e-06, 1.8e-11, 1.9e-08, and 0.12; two-sided Welch’s *t* test. **d** Mean basal surface area of each follicle cell increases at stage 7 with the transition from cell division to endoreplication. *P* values = 5.3e-05, 0.19, 0.84, 0.0005, and 1.8e-12; two-sided Welch’s *t* test. **e** Kinetics of follicle elongation. *P* values = 2.0e-06, 0.16, 0.0024, 7.8e-09, and 9.8-e03; two-sided Welch’s t-test. n, biologically independent samples. Error bars, s.e.m. NS not significant, **P < 0.01, ****P < 0.0001

Accurate morphometric comparisons are dependent upon objective identification of developmental stages. We merged classical morphological staging^11^ with quantitative data obtained above, and generally found good accordance that allowed boundaries based on follicle cell numbers to be set (Supplementary Fig. 1). Stage 6, which is critical for elongation analyses, does not have clearly defined morphological criteria. We subdivided it into stages 6 A and 6B to better capture the range of cell numbers and an inferred longer duration, as well as the accelerated rate of elongation in the latter stage (Fig. 2a, e; “Methods” section). We also limited our analysis of stage 8 follicles to the presumed youngest half, excluding the larger follicles in which the transition to squamous epithelial cells at the anterior is clearly evident.

This analysis confirmed, quantified, and refined parameters of wild-type oogenesis previously derived using other methods. When considered by stage, follicle volume grows roughly exponentially (Fig. 2b), while cell numbers in the follicle epithelium increase roughly linearly (Fig. 2c), finally reaching 862 ± 32 standard deviation (s.d) cells, similar to both early and more recent estimates^12,13^. As expected, basal surface area of epithelial cells decreased during division stages and increased after the onset of endoreplication at stage 7 (Fig. 2d). Both basal surface areas and cell heights were similar along the A–P meridian through stage 6, suggesting that cells have largely equal volumes (Supplementary Fig. 2a). However, at stage 7 basal surface area significantly increased in cells at the anterior one-third and the height of these cells decreased, suggesting the onset of transition of this population to eventually become fully squamous. Overall, we measure a roughly linear increase in elongation, with an aspect ratio increase from 1.17 at stage 4 to 1.96 at early stage 8 (Fig. 2e).

### Oriented cell divisions and polarized cell shape changes

**Fig. 3:**
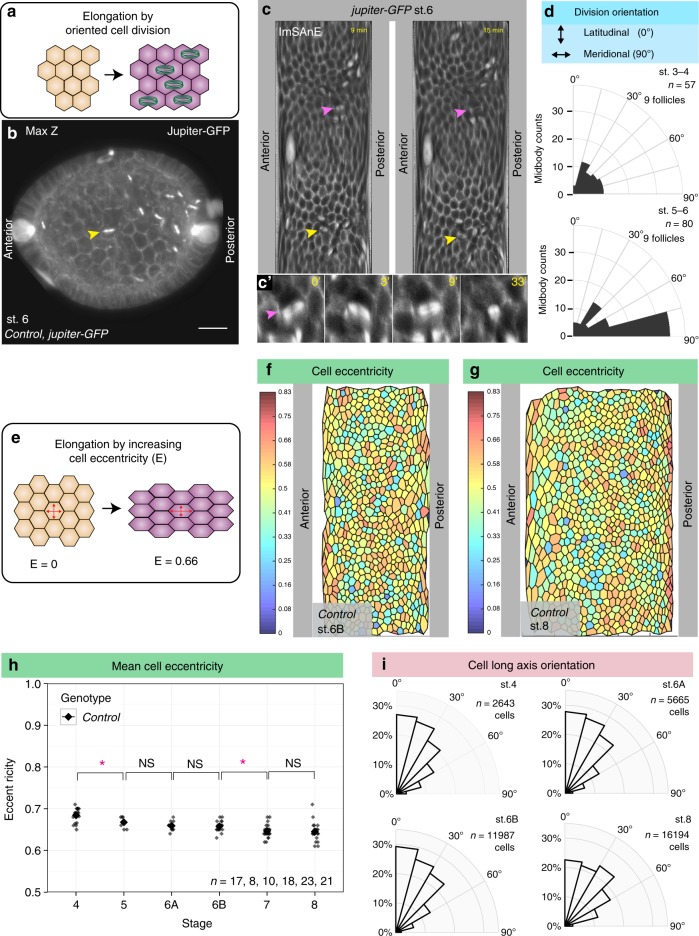
Cell division orientation and cell eccentricity in elongating follicles **a** Schematic of tissue elongation via polarized cell division. **b** Maximum Z projection from confocal stacks of live-imaged follicle expressing Jupiter-GFP (gray) to mark mitotic spindles and midbodies. **c** ImSAnE cylinder projections of the same follicle. Yellow arrowheads mark the same cell division in (**b**, **c**). Magenta arrowhead in (**c**, **c**′) marks a cell division in which the spindle changes orientation (see also Supplementary Fig. 2b). **d** Quantitation of division orientation (0° = latitudinal; 90° = meridional) shows that cytokinetic angles are broadly distributed in round stage 3–4 follicles but become oriented along the AP arc in elongating stage 5–6 follicles. **e** Schematic of tissue elongation via increased cell eccentricity. **f**, **g** ImSAnE projections of stage 6B and stage 8 follicles, color-coded for eccentricity (0 = rounded; 0.83 = highly elongated). **h** Mean cell eccentricity changes modestly during stage 4–8, as compared to the strongly increased elongation of the organ overall. *P* values = 0.015, 0.17, 0.8, 0.017, and 0.77; two-sided Welch’s *t* test. **i** Orientation of cell eccentricity (0° = latitudinal; 90° = meridional) shows that most cells are elongated perpendicular to the AP axis. *n*, biologically independent samples. Error bars, s.e.m. NS not significant, *P < 0.5. Scale bar, 10 μm

We then considered the morphogenetic behaviors that could drive follicle elongation. In other systems, tissue elongation is known to result from oriented cell division, polarized cell shape changes, or oriented cell rearrangements^13^. We analyzed follicles using ImSAnE to capture parameters for every cell, eliminating artifacts associated with the imaging plane and preserving cell orientation along the arcs of the curved epithelium, including at the poles. To analyze the orientation of cell division (Fig. 3a), we expressed the microtubule-binding protein Jupiter-GFP to mark the mitotic spindles and midbodies (Fig. 3b–c and Supplementary Movie 2); we then live-imaged follicles from stage 3 through stage 6, when mitotic divisions terminate. Midbody position, reflecting the ultimate plane of cytokinesis, was variably aligned in the epithelial plane during stage 3–4, but during stage 5–6 became preferentially aligned along the meridian (Fig. 3d). We noted that the initial orientation of the mitotic spindle was significantly less biased than the mitotic plane, even at stage 5–6. Instead, A–P alignment of the spindle often occurred after metaphase, raising the possibility that the outcome of division is organized by tissue-wide tension (Supplementary Fig. 2b–c and Supplementary Movie 3). Nevertheless, oriented cell divisions could potentially contribute to follicle elongation prior to stage 7.

To determine if polarized cell shape changes account for overall follicle elongation (Fig. 3e), we measured the eccentricity of each follicle cell; that is, the aspect ratio of its best-fit ellipse (Fig. 3f–g). During stage 5–8, even though the distribution of cell eccentricity changes, indicating that cell shapes are not static (Supplementary Fig. 2d), follicle cells maintain a quite consistent mean eccentricity, between 0.65–0.7 (Fig. 3h). Importantly, we also determined the orientation of cell eccentricity within the epithelial plane and found that at all stages, most cells’ long axes are oriented latitudinally (Fig. 3i). Cells therefore tend to be elongated perpendicular to, rather than parallel to, the axis of tissue elongation, and their average shapes change only slightly during a near doubling of organ aspect ratio.

### Cell rearrangement and cell reorientation during elongation

**Fig. 4:**
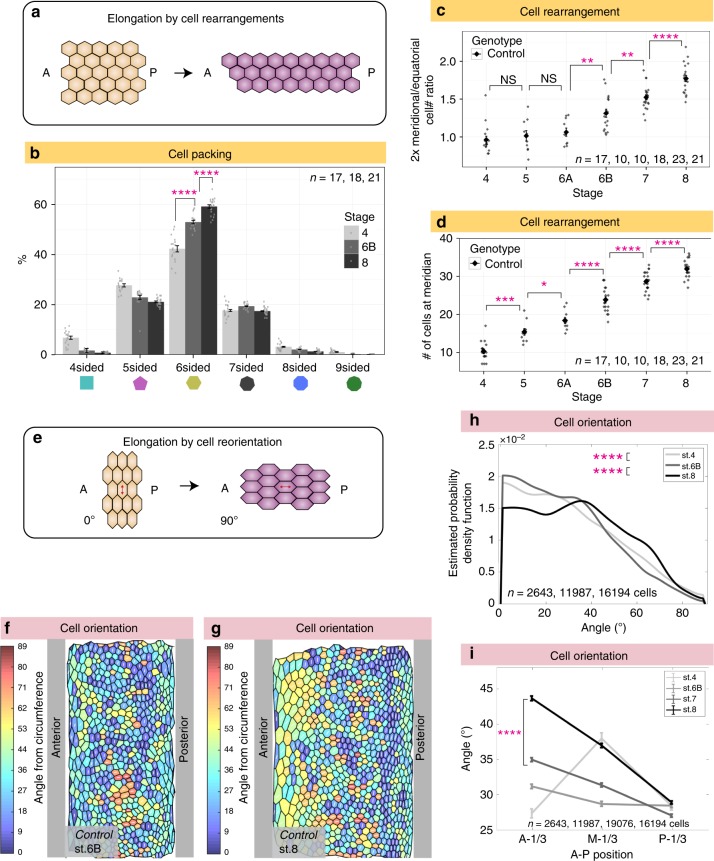
Cell rearrangements and cell reorientation in elongating wild-type follicles. **a** Schematic of tissue elongation via cell rearrangements. **b** Quantitation shows increases in topological order (i.e. fraction of hexagonal cells) from stage 4 to stage 8. *P* values = 6.7e-08 and 2.8e-07; two-sided Welch’s *t* test. *n*, biologically independent samples. **c** Cell counts along two arcs demonstrate increases in the ratio of meridional cells to equatorial cells from stage 6B through stage 8. Meridional cell number, defined in Fig. 1g, is doubled on the y axis in (**c**) to capture entire follicle perimeter, as does the equatorial cell number. *P* values = 0.55, 0.58, 0.003, 0.0024, and 2.2e-05; two-sided Welch’s *t* test. *n*, biologically independent samples. **d** Increases in the number of meridional cells also demonstrate cell intercalation. *P* values = 1.8e-04, 0.02, 7.8e-05, 2.0e-05, and 4.2e-05; two-sided Welch’s *t* test. *n*, biologically independent samples. **e** Schematic of tissue elongation via cell reorientation: anisotropically-shaped cells remodel cell junctions to shift the direction of their long axis. **f**, **g** ImSAnE cylinder projections of stage 6B and stage 8 follicles, color-coded for cell orientation (0° = latitudinal; 90° = A–P). **h** Graph showing distribution of cell orientations in wild-type follicles: estimated probability density function (*y* axis) reflects the relative frequency of cells with a given long axis orientation (*x* axis, as defined in (**e**)). From stage 4–8, the proportion of A–P-oriented cells increases significantly. *P* values = 2.0e-05 and 3.27e-83; Two-sample Kolmogorov–Smirnov test. *n* = number of follicle cells. **i** Cells in the anterior one-third of the follicle between stage 6B and 8 reorient to become increasingly A-P oriented (A-1/3: anterior one-third; M-1/3: middle one-third; P-1/3: posterior one-third; values are mean orientation for each region). *P* value = 4.22e-86; two-sided Welch’s *t* test. *n* = number of follicle cells. Error bars, s.e.m. NS not significant, *P < 0.5, **P < 0.01, **P < 0.001, ****P < 0.0001. Source data are provided as a Source Data file

To explore whether cell rearrangements (Fig. 4a) occur during follicle elongation, we first analyzed cellular topology in fixed follicles (Fig. 4b). Changes in topological order can be caused by either cell proliferation or by neighbor exchange, and ordered tissues have a greater proportion of hexagonal cells^14^. Stage 4 follicles were the most disordered, likely reflecting their high rate of cell division, rather than tissue elongation which is limited at this time. Topological order then increased, indicating formation of new cell junctions.

To seek evidence of cell rearrangements, we counted cells along the follicle arcs. These counts revealed a 65% increase in the ratio of meridian to equatorial cells from stage 6 to 8 (Fig. 4c). Moreover, in post-mitotic follicles between stages 7 and 8, an addition of ~3 epithelial cells along the A–P meridian was seen (Fig. 4d). These data demonstrate that cell rearrangements do occur during follicle elongation, leading to intercalation of cells from the equatorial to the A–P axis.

We next searched for intercalation events by examining follicles live-imaged ex vivo. We did not see rosette-like arrangements, and while we saw occasional 4-cell (T2-like) junctions^15,16^, we were unable to consistently identify T1 > T3 transitions in movies from stage 6 to 8. However, live-imaged follicles at these stages showed signs of deterioration after 6 h, only ~1/4 of the period in which elongation takes place, limiting our ability to draw conclusions from this approach.

We then analyzed cell orientation. Although the mean eccentricity of follicle cells is persistent, the shifts in cell long axis angle distribution (Fig. 3i) raise the possibility that these changes in orientation could be a mechanism for tissue elongation (Fig. 4e). Strikingly, as oogenesis proceeds, certain cells reorient their long axis, from primarily along the circles of latitude to increasingly along the A–P meridian (Fig. 4f–h). Between stage 7 and 8, reorientation was particularly evident in the anterior third of the follicle (Fig. 4i), where the average angle of orientation towards the meridian increases by 8.7°. This cell reorientation, which is coincident with a period of post-mitotic tissue elongation, is thus a candidate contributor to it.

### Cell behaviors in elongation-defective mutants

**Fig. 5:**
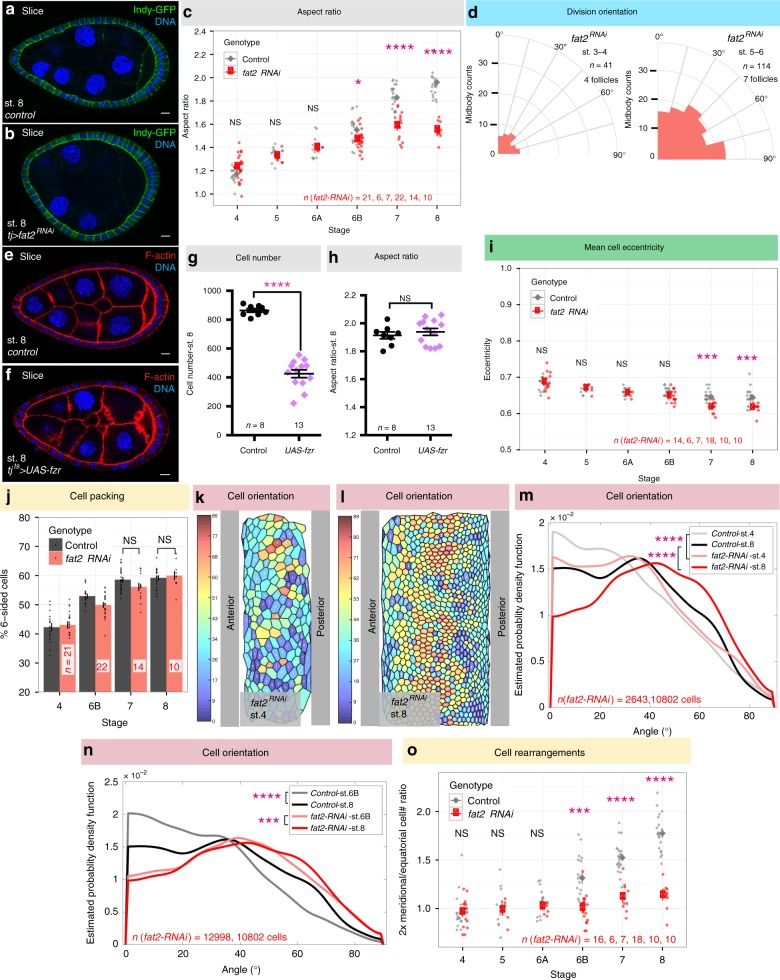
Morphometrics of elongation-deficient follicles. **a**, **b** Control (**a**) and *fat2-*depleted (**b**) follicles at stage 8. **c** Aspect ratio of *fat2-*depleted follicles diverges from wild type at stage 6B. *P* values = 0.07, 0.52, 0.89, 0.02, 1.45e-06, and 1.35e-11; two-sided Welch’s *t* test. **d** Cell divisions remain ~randomly oriented in both stage 3–4 and stage 5–6 *fat2-*depleted follicles. **e**–**h** Control (**e**) and Fzr-overexpressing (**f**) follicles at stage 8. Premature onset of endoreplication via acute expression of Fzr ~halves follicle cell number (**g**, *P* value < 0.0001; two-sided Welch’s *t* test), but does not prevent tissue elongation (**h**, *P* value = 0.49; two-sided Welch’s *t* test). **i** Cell eccentricity in *fat2-*depleted follicles does not differ from wild type until stage 7, when cells become slightly less elongated. *P* values = 0.70, 0.67, 0.73, 0.08, 2.2e-04, and 3e-04; two-sided Welch’s *t* test. **j** Topology analysis shows that cells in stage 8 *fat2-*depleted follicles can rearrange to reduce tissue disorder. *P* values = 0.10, and 0.64; two-sided Welch’s *t* test. **k**, **l** ImSAnE cylinder projections of stage 4 and stage 8 *fat2-*depleted follicles, color-coded for cell orientation (0° = latitudinal; 90° = meridional). **m**, **n** Distributions show that *fat2-*depleted follicles show aberrant orientation distributions at stage 4, and do not show the reorientation evident in wild type at stage 8. In **m**, *P* values = 1.67e + 05 and 4.0e-66; Two-sample Kolmogorov–Smirnov test. In **n**, *P* values = 3.27e-83 and 6.18e-04; Two-sample Kolmogorov–Smirnov test. **o** Cell counts along two arcs reveal that *fat2-*depleted follicles fail to rearrange cells from circumferential to meridional. *P* values = 0.92, 0.69, 0.42, 3.28e-05, 1.40e-06, and 1.55e-10; two-sided Welch’s *t* test. *n*, biologically independent samples. Scale bars, 10 μm. Error bars, s.e.m. NS not significant, *P < 0.5, **P < 0.01, ***P < 0.001, ****P < 0.0001

To test the functional role of the above cell behaviors, we analyzed them in mutant backgrounds that either altered the behaviors specifically or altered follicle elongation overall. The most frequently-used elongation mutant disrupts *fat2*, which encodes an atypical cadherin required for follicle planar cell polarity and tissue rotation^17,18^. *Fat2* mutant or RNAi-depleted follicles show discontinuously variable stiffness^8,19^, and their elongation diverges from wild-type follicles at stage 6B; by stage 8 they have aspect ratios of 1.55 ± 0.08 s.d. instead of 1.96 ± 0.13 s.d. (Fig. 5a–c). Growth of *fat2*-depleted follicles and proliferation of their epithelial cells are unchanged compared to wild type, with the exception of having 8% more cells at stage 6B and 10% at stage 8, respectively (Supplementary Fig. 3a–b). Since total surface area of *fat2*-depleted follicles remains similar to wild type (Supplementary Fig. 3c), the increase in cell numbers leads to a 11% reduction in mean basal surface area of individual cells (Supplementary Fig. 3d).

When analyzing cell division orientation in *fat2*-depleted follicles at stage 3–6, we found that, in contrast to WT, the plane of cytokinesis did not orient along the A–P meridian at stage 5–6, but remained randomly aligned as it is in stage 3–4 (Fig. 5d). Because *fat2*-depleted follicles show disrupted BM stiffness at stage 5, before defects in elongation are evident^8^, we considered the possibility that the altered divisions in these follicles result from stiffness loss, whereas the oriented divisions seen in wild type are a consequence of the stiffness gradient. Consistent with this hypothesis, follicles depleted of the major BM component Collagen IV (ColIV) also showed random cell division orientation (Supplementary Fig. 3e). Moreover, RNAi to *mushroom body defective (mud)*, which disrupts mitotic plane alignment in follicle cells^20^, did not induce elongation defects (Supplementary Fig. 3f–h). To further test whether it is oriented cell divisions that drive follicle elongation, we inhibited all mitoses in otherwise wild-type follicles by overexpressing *fizzy-related (fzr)*, a Cdh1 homolog that is sufficient to switch cells from proliferative to an endocycle program^21^. Fzr overexpression from stage 4 limited epithelial cell numbers to only 426 ± 97 s.d. cells, which is 49% of wild type (Fig. 5g). Nevertheless, these follicles elongated similar to wild type controls (Fig. 5e–h). We conclude that oriented cell divisions are not required for follicle elongation.

We then investigated cell shapes, rearrangements, and orientation changes in *fat2*-depleted follicles. Despite the fact that Fat2 is required for follicle rotation, cell eccentricity was similar to wild type from stage 4 to stage 6B (Fig. 5i), demonstrating that elongated cell shapes are not caused by collective cell migration. Cell topology at stage 7–8 also did not differ from wild type (Fig. 5j), indicating that *fat2* cells are capable of rearranging to increase tissue order. However, the initial distribution of cell orientations in *fat2* follicles differed from wild type even at stage 4: *fat2* cells orient their long axis more along the A–P meridian, with a broader distribution of orientations than the highly skewed distribution of wild type (Fig. 5k–m). This phenotype can be seen before significant differences in follicle elongation are evident and represents the earliest morphometric defect seen in *fat2* follicles. Moreover, whereas the distribution of orientations in wild-type follicles shifts substantially following stage 6B, *fat2-*depleted follicles show only a minor shift (Fig. 5n). The shift in wild-type follicles from stage 6B to 8 changes mean cell orientation 5 times the change seen in *fat2-*depleted follicles (5° vs 1°). Finally, equatorial and meridional cell counts to assess intercalation between stage 6 and stage 8 revealed that the ratio did not increase in *fat2*, as it did in wild type (Fig. 5o). These data suggest a relationship between changes in cell orientation and the cellular rearrangements that elongate the follicle.

### Rack1 negatively regulates Src to elongate the follicle

**Fig. 6:**
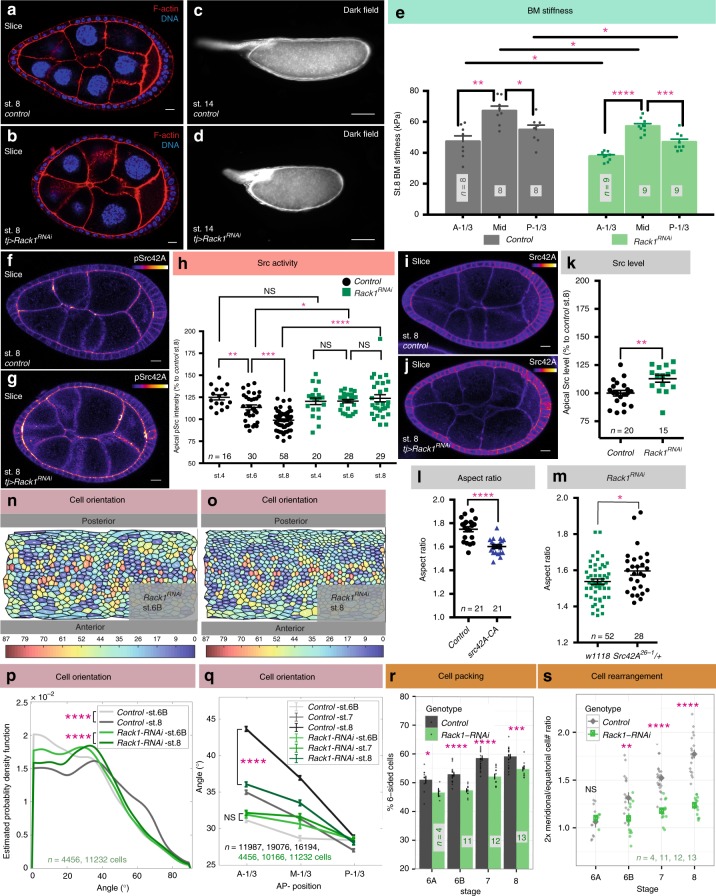
Src regulator Rack1 controls cell reorientation and follicle elongation. **a**–**d** RNAi-mediated depletion of *Rack1* produces round follicles (**b**) and round eggs (**d**) compared to controls (**a**, **c**). **e** AFM analysis of the BM of *Rack1*-depleted follicles shows preservation of an AP gradient of stiffness, although average stiffness is slightly decreased. *P* values between *control* at different positions = 0.0011 and 0.013, between *Rack1*^*RNAi*^ at different positions = < 0.0001 and 0.0009, between control and Rack1^*RNAi*^ = 0.037(A-1/3), 0.016(M-1/3), and 0.045(P-1/3); two-sided Welch’s *t* test. **f**–**h** *Rack1*-depleted follicles (**g**) show increased levels of pSrc at apical junctions compared to wild type (**f**). In (**h**), *P* values between *control* stage-4 and 6, stage-6 and 8 = 0.007 and < 0.0001; between Rack1^RNAi^ stage-4 and 6, stage-6 and 8 = 0.96, 0.47; between *controls* and Rack1^RNAi^ *s* = 0.34, 0.04, < 0.0001; two-sided Welch’s *t* test. **i**–**k** Levels of apical Src42A are increased in Rack1-depleted follicles. *P* values = 0.0031; two-sided Welch’s *t* test. **l** Hyperactivation of Src42A impairs follicle elongation. **m** Heterozygosity for Src42A partially suppresses elongation defects of *Rack1*-depleted follicles at stage 8. **n**, **o** ImSAnE cylinder projections of stage 6B and stage 8 *Rack1-*depleted follicles, color-coded for cell orientation (0° = latitudinal; 90° = AP). **p** Distributions of cell orientation in wild type and *Rack1-*depleted follicles. *P* values = 3.27e-83 and 8.97e-06; Two-sample Kolmogorov–Smirnov test. *n* = number of follicle cells. **q** Cells in the anterior third of *Rack1-*depleted follicles fail to reorient towards the AP. *P* values between stage 6B control and *Rack1*^RNAi^ = 0.18 (A-1/3), 0.004 (M-1/3), and 0.91 (P-1/3); two-sided Welch’s t test. *P* values between stage 8 control and *Rack1*^RNAi^ = 1.51e-58 (A-1/3), 8.31e-13 (M-1/3), and 0.01 (P-1/3); two-sided Welch’s *t* test. *n* = number of follicle cells. **r** *Rack1-*depleted follicles impair to rearrange cells to reduce tissue disorder. *P* values = 0.046, 1.6e-06, 9.3e-09, and 0.0001; two-sided Welch’s *t* test. **s** *Rack1-*depleted follicles fail to rearrange cells from the equatorial to meridional. *P* values = 0.66, 0.00962, 2.67e-08, and 2.10e-12; two-sided Welch’s *t* test. Error bars, s.e.m. Except for (**p**, **q**), *n*, biologically independent samples. NS not significant, *P < 0.5, **P < 0.01, ***P < 0.001, ****P < 0.0001. scale bars in (**a**, **b**, **f**, **g**, **i**, **j**), 10 μm and in (**c**, **d**), 100 μm. Source data are provided as a Source Data file

The mechanically patterned BM that is proposed to sculpt the tissue^8^ is disrupted in the *fat2-*depleted follicles analyzed above. To uncover how elongation-driving cell behaviors normally respond to these ECM cues, we searched for mutants that are defective in elongation but nevertheless retain graded BM stiffness. We exploited an ongoing genetic screen in the lab in which transgenic RNAi lines are expressed specifically within the follicle epithelium, and those that perturb oogenesis are scored as hits. One hit targeted *Receptor for Activated Protein Kinase C 1 (Rack1)*, which gave rise to round follicles and round eggs with a high degree of penetrance (Fig. 6a–d). Elongation defects emerged at stage 6B, similar to *fat2*-depleted follicles (Supplementary Fig. 4a). This phenotype was seen with two independent RNAi lines targeting different regions of the transcript, and was rescued by overexpression of a Rack1-encoding transgene (Supplementary Fig. 4b-d). It was further confirmed by making mitotic clones of *Rack1* null cells in the epithelium, where moderately-sized clones altered follicle aspect ratio (Supplementary Fig. 4e–g). Thus, Rack1 is a regulator of egg elongation.

Analysis of *Rack1*-depleted follicles revealed many similarities to wild type, along with modest differences in several assays. Growth appeared slightly accelerated during stage 6B and 7 (Supplementary Fig. 4h-i), and *Rack1-*depleted follicles displayed the increase in stage 8 cell number also seen in elongation-defective *fat2* (Supplementary Fig. 4j). In ex vivo culture, *Rack1*-depleted follicles rotated with normal speed and trajectory (Supplementary Fig. 4k and Supplementary Movie 4). A reporter for signaling through the JAK-STAT pathway, which is required for elongation^8,^^22^, displayed wild type-like pattern at both poles (Supplementary Fig. 4l-n). At stage 6, cell divisions were oriented along the elongation axis, comparable to wild-type follicles (Supplementary Fig. 4o).

We then assayed BM mechanics in *Rack1*-depleted follicles. Direct Atomic Force Microscopy (AFM) measurements of the BM showed a modest reduction in overall stiffness, but A–P anisotropy remained, as in wild type (Fig. 6e). This contrasts strongly with the disorganized stiffness of *fat2* follicle BMs^8,19^. When challenged with osmotic pressure induced by placement in distilled water, *Rack1*-depleted follicles burst slightly more frequently than wild-type follicles, but not as rapidly as *fat2* follicles^8^ (Supplementary Fig. 4p); bursting position also more closely resembled wild type. The overall mechanical properties of *Rack1*-depleted follicles resemble those seen in follicles overexpressing SPARC or carrying *fat2* hypomorphic alleles that delete only the intracellular domain of the protein, yet these latter genotypes elongate normally, while *Rack1*-depleted follicles do not^8,^^23^. This contrast suggests that Rack1 could be required for cellular behaviors that are triggered in response to the BM stiffness gradient.

Rack1 is a scaffolding protein whose seven WD-40 domains interact with the Src tyrosine kinase, maintaining it in an inactive state^24^. We asked whether Rack1 might negatively regulate Src activity during follicle elongation. The *Drosophila* genome encodes two Src orthologs, Src42A which is expressed in follicle cells, and Src64B which is not^25,26^. In wild-type follicle epithelia, active phosphorylated Src42A (pSrc) was found primarily at adherens junctions (AJs) (Fig. 6f). AJ staining of pSrc was significantly elevated in *Rack1*-depleted follicles starting at stage 6 (Fig. 6g–h); apical Src42A levels also showed elevation (Fig. 6i–k). Overexpression of active Src42A was sufficient to induce elongation defects in otherwise wild-type follicles (Fig. 6l), while reducing Src42A activity via heterozygosity for a null allele ameliorated the elongation defect in *Rack1-*depleted follicles (Fig. 6m).

### Src-mediated junctional remodeling reorients cells

The above results suggest that *Rack1* loss and associated Src activation alters morphogenetic cell behaviors. To identify these behaviors, we analyzed cell and junctional morphology in *Rack1* follicles. While cell eccentricity from stage 6 to stage 8 was slightly higher than wild type (Supplementary Fig. 5a), the strongest defects were seen in cell orientation. At stage 6B, mean cell orientation in *Rack1*-depleted follicles was only 1° different than WT, as compared to the 10° difference seen in *fat2*-depleted follicles (*p* value = 0.006 versus 2.7e-307; Two-sided Welch *t* test). The change in distributions at this stage was also much less significant in *Rack1* vs WT compared to *fat2* vs WT *(p* value *=* 0.0002 versus 4.8e-231; Two-sample Kolmogorov–Smirnov test) (Fig. 6n, p). However, the reorientation of cells seen during stages 7–8 in wild type was notably impaired in *Rack1*-depleted follicles (Fig. 6o–p). In particular, the anterior cells that realign towards the A–P axis in wild type fail to do so in *Rack1*-depleted follicles, remaining more latitudinally aligned (Fig. 6q). In contrast to wild type and *fat2*, *Rack1* depletion also did not resolve tissue disorder as reflected in cell topology, as *Rack1-*depeleted follicles showed lower percentages of hexagonal cells at stage 8 (Fig. 6r). Finally, cell counts along the follicle arcs established that *Rack1*-depleted follicles fail to intercalate cells along the A–P meridian (Fig. 6s).

**Fig. 7:**
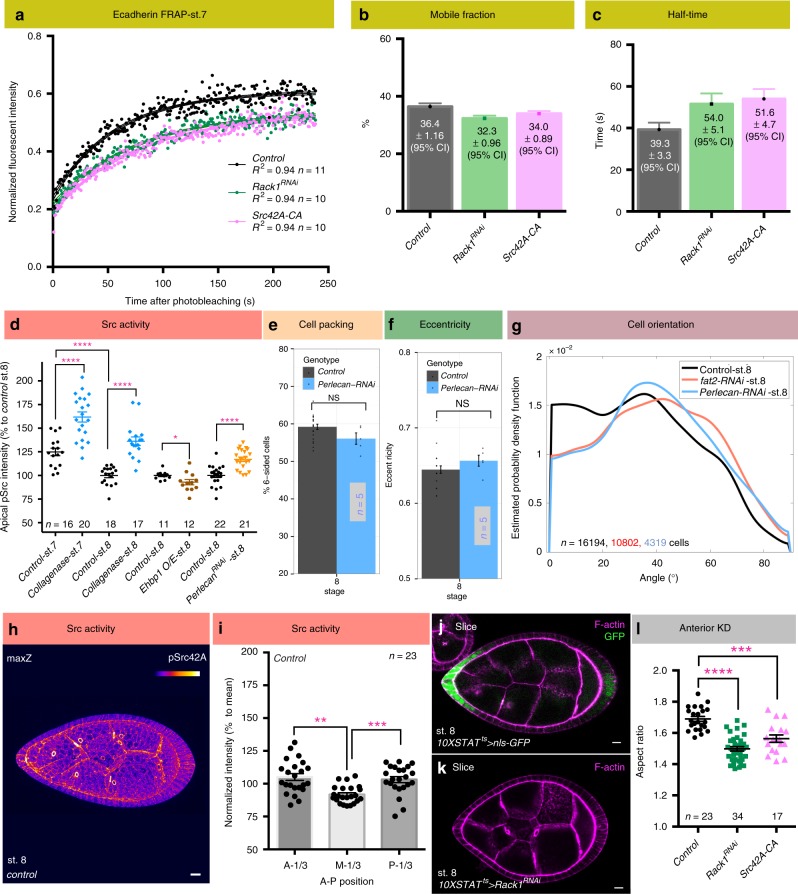
BM stiffness-responsive Src regulates AJ dynamics. **a** FRAP analysis of Ecad-GFP in *control*, *Rack1-*depleted, and *Src42A* hyperactivated follicles at stage 7. Dots represent mean of normalized intensity after photobleaching and lines are fitted curves with error bars showing 95% confidence intervals (see also Supplementary Movie 5). **b**, **c** Mobile fraction of Ecad-GFP is similar in all three genotypes, but recovery half-time are both increased when *Rack1* is depleted or *Src42A* is hyperactivated. Error bars, 95% confidence intervals. **d** Quantitation of pSrc levels shows that reduced BM stiffness is associated with increased Src activity (*control* stage 7 vs. stage 8, collagenase treatment, and depletion of Perlecan; all *P* values < 0.0001; two-sided Welch’s *t* test), while increased BM stiffness in Ehbp1 overexpressing (O/E) follicles have slightly decreased Src activity (*P* values = 0.037; two-sided Welch’s *t* test). **e**, **f** Cell topology and eccentricity of stage 8 *Perlecan*-depleted follicles resembles wild type. **g** Cell orientation distribution in stage 8 *Perlecan*-depleted follicles resembles *fat2*.; *n* = number of follicle cells. **h** Maximum Z projection of a wild-type stage 8 follicle shows differential pSrc levels along the A–P meridian. **i** Quantification of pSrc intensity in wild-type stage 8 follicles along the A–P meridian (A-1/3: anterior one-third; M-1/3: middle one-third; P-1/3: posterior one-third). pSrc intensities were normalized to mean pSrc levels of each follicle. *P* values = 0.001, 0.0004; Two-sided paired sample *t* test. **j** *10XSTAT-GAL4* drives transgene expression predominantly in follicle anterior at stage 7–8, where BM is soft and cell reorientation takes place. **k**, **l** Depletion of *Rack1* as well as Src hyperactivation in the follicle anterior is sufficient to induce elongation defects. *P* values, < 0.0001, = 0.0001; two-sided Welch’s *t* test. Error bars in (**d**–**f**, **i**, **l**), s.e.m. Except for (**g**), *n* biologically independent samples. NS not significant, *P < 0.5, **P < 0.01, ***P < 0.001, ****P < 0.0001. Scale bars, 10 μm. Source data are provided as a Source Data file

Because Rack1 and Src have been implicated in cell junction remodeling in other systems^24,25,^^27^, we analyzed junctional dynamics by performing Fluorescence Recovery After Photobleaching (FRAP) on stage 7 follicles expressing natively GFP-tagged E-cadherin (Ecad-GFP)^28^ (Fig. 7a and Supplementary Movie 5). The follicle equator was first used as the FRAP site because it provided a consistent location between samples. While steady-state levels of Ecad-GFP did not differ between wild type and *Rack1*-depleted follicles, FRAP analysis revealed changes in dynamics. No differences in the mobile fraction were seen between the two genotypes (Fig. 7b), but recovery was significantly slowed in *Rack1*-depleted follicles, with a ~40% increase in the recovery half-time (Fig. 7c). Recovery in both genotypes after photobleaching was not due to lateral diffusion (Supplementary Fig. 5b). Importantly, FRAP of follicles expressing constitutively active Src42A showed nearly identical Ecad dynamics (Fig. 7a–c). Taken altogether, these data are consistent with a model in which Rack1-limited Src42A activity regulates Ecad trafficking to permit junctional rearrangements that elongate the tissue.

### Src activity is responsive to BM mechanical cues

We then investigated the relationship between Src activity, cellular orientation, and BM stiffness. Src is considered a mechanotransducer^29^, and interestingly pSrc levels decrease as follicles develop, anticorrelating with increasing BM stiffness (Fig. 6h and Fig. 7d). Moreover, acute collagenase treatment caused a significant increase in pSrc (Fig. 7d and Supplementary Fig. 5c, d), suggesting that cellular Src activity could be negatively regulated by BM stiffness. To test this proposition further, we analyzed elongation-defective follicles depleted of the ECM component Perlecan, which undergo rotation but display a uniformly soft BM (Supplementary Fig. 4k)^19^. Indeed, *Perlecan*-depleted follicles also showed significant increases in pSrc levels (Fig. 7d and Supplementary Fig. 5e-f), while increasing BM stiffness by overexpression of EHBP1^30^, which enhances Collagen IV fibril formation^31^, had the opposite effect (Fig. 7d and Supplementary Fig. 5g, h). *Fat2-*depleted follicles displayed increased variation in pSrc levels, consistent with their variable BM stiffness^8,19^ (Supplementary Fig. 5i). Morphometric analysis showed that cell topology and eccentricity of *Perlecan*-depleted follicles resembled wild type (Fig. 7e–f), but cell orientation at stage 8 was defective, displaying a distribution more similar to that seen in follicles depleted of *fat2* (Fig. 7g). These data suggest that the BM stiffness could regulate cellular orientation changes through negative regulation of Src activity.

Finally, quantitation of pSrc levels along the A–P meridian in wild-type follicles at stage 8 revealed higher Src activity both at the anterior and the posterior than in the middle, suggesting a Src activity gradient reciprocal to the BM stiffness gradient (Fig. 7h–i). This gradient was flattened in *Rack1*-depleted follicles, although elevation at the posterior remained significant (Supplementary Fig. 5j). To investigate why cell reorientation is limited to the follicle anterior, we conducted FRAP of Ecad-GFP at both anterior and posterior regions (Supplementary Fig. 6a, b). Although these experiments were hampered by variability in FRAP sites between appropriately oriented follicles, the data indicate that higher pSrc levels in the anterior compared to the equator did correlate with increased half-time, and the difference between anterior and equator was eliminated in *Rack1-*depleted follicles (Supplementary Fig. 6c, d). By contrast, the half-time at the posterior appeared very similar to that at the equator, and this parameter, like pSrc levels, was relatively insensitive to Rack1 presence, perhaps because of differences in anterior and posterior follicle cell fates (see “Discussion” section). If BM stiffness indeed regulates Src-mediated changes in Ecad dynamics and cell orientation, then Rack1 should be required for follicle elongation primarily at the anterior, an area of soft BM that is the predominant site of reorientation. To test this, we created a GAL4 driver (10XSTAT-GAL4) that is preferentially expressed in anterior follicle cells at stages 7–8 (Fig. 7j and Supplementary Fig. 6e). Consistent with the model, RNAi-mediated depletion of Rack1 via 10XSTAT-GAL4 was sufficient to generate elongation defects, with a severity comparable to depletion throughout the follicle epithelium (Fig. 7k, l). Overexpression of active Src42 with this anterior driver also was sufficient to generate elongation defects (Fig. 7l). Together, these data indicate that differences in the mechanical status of the BM, signaled via Src, regulate anterior cell reorientation to allow tissue elongation.

## Discussion

Mechanical regulation of animal tissues is a current frontier of biology, as quantitative tools and model systems allow interrogation of unappreciated phenomena^32–35^. An emergent model for morphogenesis shaped by extracellular as well as intracellular forces is the *Drosophila* follicle. Here we use in toto image analysis to define the cell dynamics that elongate this organ and to probe their regulation by mechanical properties of the extracellular matrix. We find that polarized reorientation of a subset of cells, involving the shift of their long axes from latitudinal towards A–P meridional, is a major driver of elongation. This process fails when BM mechanics are compromised, and is regulated by the Src tyrosine kinase, which controls remodeling of cell–cell junctions. This unusual mechanism highlights differences between the elongation of bounded tissues and edgeless acinar epithelia.

All three morphogenetic behaviors that are engines for tissue elongation in other systems (cell shape changes, cell division, and cell rearrangement) are seen during follicle elongation. Average cellular elongation changes little during follicle elongation, and an initial cell shape anisotropy–lengthening around the organ circumferential axis–is not perturbed in a round egg mutant. Additionally, tissue rotation alone is insufficient to direct elongation-driving cell dynamics. Our manipulations show that oriented cell division is not essential for elongation, and its absence can be compensated by other polarized cell behaviors, as also seen in e.g. the pupal thorax^36^. Moreover, addition of cells to the elongating axis of the follicle continues at stage 7, after all mitoses have ceased. Instead, our data suggest that the major tissue-elongating behavior involves changes in cell orientation. In particular, cells in the follicle anterior shift the direction of their long axis towards the A–P axis. Without changing average cellular elongation, this reorientation of anisotropically-shaped cells changes neighbor relationships; we suggest that this results in net cellular intercalation along the A–P axis.

The number of frank intercalations that occur during follicle elongation appears relatively limited. For instance, of the ~850 cells in a post-mitotic stage 7 follicle, only ~3 are added to the meridional arc during ~8 h prior to stage 8, inducing a ~10% change in aspect ratio. This relative paucity of intercalation events contrasts not only with the rapidly developing *Drosophila* germband, but also with other elongating tissues such as the *Drosophila* pupal wing and thorax and the vertebrate neural tube and mesoderm^37^. Three major differences bear consideration here. First, significant growth (~9-fold) of both epithelial cells and the follicle overall occur during the 24–34 h^11,38^ that span stages 4 to 8, while the other systems largely rearrange a constant tissue volume. Second, the other systems have defined boundaries to impose vectorial orientation of cell behaviors, while the topologically continuous follicle epithelium lacks a boundary in the equatorial axis. Third, follicle elongation does not require conventional PCP morphogenetic signaling, nor is there evidence of PCP Myosin localization that remodels cell junctions^8,39^. Instead, the instructive force seems to be patterned anisotropic resistance to growth^8^, wherein softer BM at the poles triggers regional changes in cell behavior. Indeed, the largest changes in follicle cell orientation are seen at the anterior pole, and genetic manipulation in this region alone can prevent elongation. It is important to note that our data do not identify BM stiffness differences between the anterior and the posterior prior to cell orientation changes in the former, nor differences in pSrc levels, but do identify differences in Ecad dynamics in the latter. We speculate that A–P patterned fates in the follicle epithelium prevent elongation behaviors in the posterior, consistent with the observation that follicles lacking posterior fate specification elongate at both poles^40,41^. Overall, these differences with bounded epithelia undergoing elongation via convergent extension emphasize the new perspectives required for analysis of elongating tubular organs.

How do cells sense BM stiffness to change their orientations? The elongation-defective phenotype of *Rack1*-depleted follicles points to one mechanism involving Src. Rack1 is a Src-inhibiting protein, and its loss, with associated increases in cellular Src activity, perturbs follicle elongation. In *Rack1*-depleted follicles, initial cell orientation is not greatly altered, but anterior follicle cells remain static and are unable to shift their orientation during stages 6–8. This suggests that proper regulation of Src is required for the dynamic reapportionment of cell shape that is reflected in this switch. One familiar regulatory target of Src is integrins and their associated proteins^42,43^, but no defects in integrin-dependent cell migration^9^ are seen when Rack1 is depleted from follicle cells. However, Src has also been implicated in directly regulating AJ remodeling through effects on Ecad trafficking^44–46^, and FRAP analysis reveals compromised Ecad dynamics when follicles cells lack Rack1. Src is considered a mechanotransducer, and pSrc levels anticorrelate with BM stiffness in wild type, mutant and manipulated follicles. We therefore propose that Src mediates the instructive cue provided by BM stiffness, inducing AJ remodeling to drive morphogenesis. Whether the AJ-regulating apical Src seen in follicles is directly phosphorylated by basal integrin activation, or results from an indirect intracellular signaling cascade, remains to be investigated.

The defective tissue topology and reduced Ecad mobility seen in *Rack1*-depleted follicles suggests that Src is required for both elongation-driving and tissue disorder-minimizing cell rearrangements. This role of Src in follicle elongation raises interesting parallels with a second edgeless epithelium: the *Drosophila* trachea. In this established tubulogenesis model, gain as well as loss of Src42A activity results in shortened but broader tubules, which result from inappropriately oriented cells^47,48^. Interestingly, depletion of Src42A from the follicle, like its activation, also induced elongation defects (Supplementary Fig. 6f, g and Supplementary Fig. 3h), while Src controls tracheal Ecad dynamics^27^, perhaps in response to an ECM, albeit apically localized^47,48,49^. Thus, in both organs Src42A could mediate stiffness-cued AJ dynamics that change the orientation of cell eccentricity. Since mammalian kidney tubule development also shows Src-dependence^50^, these results suggest that ECM-mediated control of cell junctions via Src may be a general mechanism for morphogenesis of edgeless epithelia.

The data described in this work stem from a comprehensive analysis of follicle morphogenesis, which was enabled by ImSAnE software. ImSAnE allowed identification of critical cell dynamics near the follicle poles, which are most subject to distortion from conventional analyses, and distinguished the cellular basis underlying overtly similar elongation phenotypes. For instance, it revealed that under *fat2* depletion, cells throughout the follicle are defective in planar polarized cell orientation from early stages, whereas in *Rack1* follicles defective orientation is limited to the anterior and occurs only later, when elongation behaviors initiate. Furthermore, some of the ImSAnE-based morphometric findings are concordant with those recently reported based on conventional imaging^51–53^. This work focuses on stages 4 through 8, and does not address cell dynamics that elongate the follicle prior to and following these stages^22,54^. However, it does provide a framework for true in toto analysis of this simple organ, at multiple developmental stages and genotypes. As genetic screens (this work,^55^) and biophysical studies^8,19,56^ in the follicle are extended, the quantitative imaging platform reported here will provide a bridge towards mathematical and mechanical modeling of this flourishing system for in toto organ morphogenesis.

## Methods

### Fly strains and husbandry

The following *Drosophila* strains were obtained from Bloomington stock center: *Jupiter-GFP*^57^ (Flybase ID: FBst0006836), *mCherry-RNAi (control)* (Flybase ID: FBst0035785), *Fat2-RNAi* (Flybase ID: FBst0040888), *Rack1-RNAi* (Flybase ID: FBst0034694), *Perlecan-RNAi* (Flybase ID: FBst0029440), *Mud-RNAi* (Flybase ID: FBst0035044), *Col4a1(Cg25c)-RNAi* (Flybase ID:*FBst0044520)*, *Src42A-RNAi* (Flybase ID: FBst0044039), *Rack1*^*1.8*^*-FRT40A*^58^, *UAS-Src42A-CA* (Flybase ID: FBst0006410), *P{tubP-GAL80*^*ts*^*}20* (Flybase ID: FBst0007019), *UAS-mcherry-Ehbp1*(Flybase ID: FBst0067145)^30^, and *w1118* (Flybase ID: FBst0003605). *Col4a2(Vkg)-RNAi* (VDRC ID: 106812, Flybase ID: FBst0478636), *Src42A-RNAi* (VDRC ID: 100708, Flybase ID: FBst0472581) were obtained from VDRC stock center. *UAS-fzr*^21^ is a gift from Brian Calvi; *Src42A*^*26–1*^ is a gift from Greg Beitel^25,48^; *UAS-Rack1.ORF* is from FLY-ORF # F001448; *Ecad-GFP* is a gift from Yang Hong^28^; *GR1-GAL4*, *UAS-FLP* is a gift from Trudi Schüpbach; *Indy-GFP* (Flybase ID: FBst0050860) *and vkg-GFP* (Flybase ID: FBti0153267) are from Flytrap^59^, *yw* is from Tom Neufeld, and *traffic jam-Gal4 (tj-GAL4)* from Kyoto Stock Center (Flybase ID: FBtp0089190). *10XSTAT-GAL4* was created by synthesizing a 2250 bp fragment (ThermoFisher, see Supplementary Methods for the sequence of the fragment) containing five tandem copies of the Socs36E enhancer^60^ and cloning into pAttB-Gal4. Detailed genotypes used in each figure are listed in Supplementary Table 1.

Adult flies were maintained at 25 °C unless otherwise noted. Adult females were flipped onto fresh food daily for 1–2 days and were fed with yeast paste overnight before dissection.

*Fat2* follicles are depleted with RNAi; their phenotype parallels molecularly characterized null alleles^8^. *Mud*, *Src42A, Collagen IV* and *Perlecan* RNAi are validated by phenocopy of strong loss of function mutants^19,61–63^. *tj-GAL4, P{tubP-GAL80*^*ts*^*}20; UAS-fzr* (*tj*^*ts*^ *>* *UAS-fzr*) along with control follicles used in Fig. 5e–h were shifted to 29° for 17.5 h before analysis. *tj*^*ts*^ follicles used in Supplementary Fig. 3l-n were shifted to 29° for 25 h before analysis. *tj*^*ts*^ *>* *Rack1-RNAi* follicles used in Fig. 6m were shifted to 29° for 24 h before analysis. *10XSTAT-GAL4; P{tubP-GAL80*^*ts*^*}* follicles used in Fig. 7j–l and Supplementary Fig. 7d were shifted to 29° for 25 h before analysis.

### Follicle staging

To objectively stage follicles, we compared morphological criteria^5^ to our quantitative morphometric data on follicle volume and epithelial cell number (Supplementary Fig. 1). For stages with definitive morphological criteria, we generally found good accordance with our data, allowing consistent boundaries based on follicle cell number to mark the end of stage 4, 7, and 8. However, stage 5 and 6 are not clearly distinguished by morphology, and there is debate about whether cell proliferation ends prior to stage 6 or continues during this stage^11,64,65^. We found that cell counts of follicles deemed stage 6 by the criteria of Jia et al.^65^ contained well fewer than 900 cells. Moreover, the frequency of such follicles in vivo (our results and ref. ^38^) is higher than would be expected for standard estimates of stage 6 duration^11^. These standard estimates of developmental stages are based on transplant of a single germarium into the abdomen of an *ovoD1* female host, and are likely faster than follicles in an entire native ovary in vivo^66^. Using 9.6 h as the average length of a follicle cell cycle^67^, the durations of stage 4, 5 and 6 based on our epithelial cell counts are 9.6, 4.8, and 8.4 h respectively, in reasonable agreement with the durations suggested based on relative follicle representation in wild type hosts^38^. Due to the wide ranges in volume observed in classically defined stage 6 follicles, and the accelerated rate of elongation in larger follicles of this group, we divided them into substages 6 A and 6B choosing 650 cells as a midpoint boundary. We also assessed convenient staging parameters in single confocal cross-sections when complete 3D follicles are not imaged. Like Dai et al.^68^, we found that nurse cell nuclear diameter allowed reasonable staging, and our measurements are close to theirs. Jia et al.^65^ used cross-sectional area of follicles, albeit with values discrepant with our data likely due to differences in mounting and imaging; we found that this parameter did not reliably distinguish follicles of different stages as determined by cell number and morphological criteria.

### Immunostaining and Imaging

Immunostaining and imaging were executed as previously described^7,23^. Antibodies used are listed in Supplementary Table 2. Phospho-Src42A (Tyr400) antibody is a gift from Shigeo Hayashi^27^ and Src42A antibody is a gift from Tetsuya Kojima^25^. Ex vivo follicle culture was performed as previously described^8^, with osmolarity adjusted to 260 Osm/L. Fluorescent images were acquired on Zeiss LSM700 confocal microscope with LD C-Apochromat 40 × /1.1 W Corr objective. Imaging for cell division orientation was performed using selective plane illumination microscope (SPIM) Zeiss LightSheet.Z1 with illumination lens 10 × /0.2 and Lightsheet Z.1 detection optics 20 × /1.0 W. Follicles were manually dissected and embedded in 0.5% low melting point agarose with complete media contained in a 0.6 mm in diameter glass capillary (Brand GmbH). Mounted follicles were immersed into a chamber filled with media without insulin supplement.

### Morphometrics extraction

We used ImSAnE to unroll the follicle epithelium as previously described^7,10^, with all measurements computed using the metric tensor. Follicle A–P orientation was defined either automatically by the longest axis or manually based on the position of the polar cells identified by anti-FasIII staining at the stages when follicles are close to spherical. Length along the A–P meridian was computed by determining the anterior and posterior poles as extreme points along the A–P axis, and then measuring the length of the geodesic connecting these two points. latitudinal length was computed by choosing an arbitrary point midway along the meridian and measuring the length around the circumference passing through this point. Follicle aspect ratio is defined as the ratio of A-P length to the length of future dorsoventral (DV) axis. Aspect ratios were computed from unflattened 3D stacks, except for Figs. 6l, 7l, Supplementary Fig. 3h, 4d, g, which used single confocal sections of preparations with standard mounting^8^. Follicle volume was computed by adding up all pixels contained within the 3D surface. The number of cells along the A–P meridian and the equatorial circumference was determined by first segmenting all cells in a pullback. Cells whose centroids are a given distance away from the line used to measure the meridional and latitudinal length were then counted accordingly. For fusing 3D representations of the surface, pullbacks showing anterior, posterior, and two cylinder projections were first segmented, and then stitched together in 3D. The pullbacks were based on a maximum intensity projection of 3 × 0.5 μm thick pullbacks from 3 to 4 μm inward of the basal-most surface extracted. Cell segmentation used simple thresholding of prediction maps obtained by using Ilastik^69^. The thresholded images were treated with standard morphological operations to obtain skeletonized cell outlines. Automated methods for detecting branching points then enabled construction of a lattice. The lattice consists of a lookuptable, with vertices, bonds connecting vertices, and cells arranging bonds (cell–cell interfaces) to a closed outline. The number of neighbors in each cell was determined by the number of bonds. Surface area was obtained by integrating the square root of the metric tensor over the cell. Summing all individual cell areas gave total follicle surface area. Cell eccentricity is determined based on the long and short axis of the cell, while cell orientation is defined as the angle between the long axis of the cell and the circumferential axis of the follicle.

### BM Stiffness Assays

Basement membrane stiffness was measured by Atomic Force Microscopy (AFM)^8^. Briefly, live follicles were indented four times using a pyramidally-tipped cantilever of calibrated spring constant mounted on a Bruker Catalyst AFM, with an approach velocity of 0.4 μm/sec and a setpoint force of 1nN. Young’s Modulus of elasticity was calculated by fitting the cantilever deflection versus piezo extension curves to the modified Hertz model, using only the first 50 nm of indentation to isolate elasticity from the BM.

### Fluorescence Recovery After Photobleaching (FRAP) analysis

FRAP was performed with Zeiss LSM 700 with LD C-Apochromat 40 × /1.1 W Corr objective at room temperature (23.3 °C). Follicles were dissected and embedded in 0.5% low melting point agarose with complete media^8^ in a glass bottom dish. Stalks at both ends of stage 7 follicles were severed for equatorial FRAP or positioned to allow stochastic alignments for either anterior or posterior FRAP ~4–8 cell diameters away from polar cells. Pre- and post-bleaching images were captured every 1 s for 5 min with a pixel resolution of 512 × 269 pixels (0.16 μm/pixel) and scan time 821.67 msec. An elliptical region of interest (ROI) ranging from 4.8 to 7.7 μm^2^ covering circumferentially oriented junctions was bleached twice by 488 nm (10 mW) solid-state laser (70% laser power for equatorial FRAP or 80% for anterior and posterior FRAP) with pixel dwell time 100.85 μs. Both drifts in time lapse images and follicle rotation were corrected and reregistered with Fiji plugin StackReg followed by manual inspection and correction for each timeframe (*t*). Intensity of ROIs including photobleached (*Y*_BL_), background (*Y*_BG_), and reference regions (*Y*_REF_) were measured using Fiji Mean gray value measurements. Normalized post-bleaching intensities (*Y*) for correcting background and photobleaching resulted from imaging were calculated by Eq. (1).

1 $$Y\left( t \right) = \frac{{Y_{{\rm{BL}}}\left( t \right) - Y_{{\rm{BG}}}\left( t \right)}}{{Y_{{\rm{REF}}}\left( t \right) - Y_{{\rm{BG}}}\left( t \right)}} \times \frac{{Y_{{\rm{REF}}0} - Y_{{\rm{BG}}0}}}{{Y_{{\rm{PRE}}} - Y_{{\rm{BG}}0}}},$$

where *Y*_*PRE*_ is pre-bleached intensity of ROI, *Y*_BG0_ is pre-bleached background intensity, and *Y*_REF0_ pre-bleached reference intensity. Recovery curves were fitted by Prism with Eq. (2) for one phase association.

2 $$f\left( t \right) = Y_0 + \left( {P - Y_0} \right) \times (1 - e^{ - Kt}),$$

where *Y*_0_ is the *Y* value immediately post bleaching, *P* is the *Y* value at infinite times, *K* is the rate constant, half-time is ln(2)/*K*, mobile fraction is *P*−*Y*_0_.

### Fluorescence intensity and follicle velocity measurements

Fiji^70^ was used to measure the intensity of apical Src and phosphorylated Src (pSrc) intensity. A 10-pixel thick line along the apical membranes was drawn with freehand line tool, followed by mean gray value measurements. Intensities were normalized based on the mean intensity of control stage 8 follicles from the same batches under the same imaging settings. Follicle rotation velocities were measured by the circumferential displacement of plasma membranes visualized with Indy-GFP at the equator.

### Collagenase treatment

ex vivo cultured follicles were treated with collagenase as previously described^8^, followed by 3× washes with Schneider’s media and 35 min of incubation at 25 °C before fixation.

### Data presentation

Opacity projection was generated by Volocity 5 (PerkinElmer). ImSAnE surface 3D projection was generated by plotting cylinder projection onto fitted 3D structure. Max Z projection was generated by Fiji Z projection-Max intensity function. Data were analyzed and charts displayed using MATLAB 2015a and 2018a (Mathworks), Excel (Microsoft), Prism 6 (Graphpad) and ggplot2 in RStudio. Figures were assembled with Adobe Illustrator CC 2018 (Adobe).

### Statistics and reproducibility

Neither sample size estimate, randomization of samples or blinding was performed. Data normality and variation estimation were not performed. Unless noted, error bars in charts represents standard error of the mean (s.e.m). Statistical significance assembled was assessed by two-sided Welch’s unequal variances *t*-test, while pSrc intensity at different A–P positions within the same follicle were analyzed by two-sided paired sample t-tests, and two-sample Kolmogorov–Smirnov test was used to analyze estimated probability distribution functions in cell eccentricity and cell orientation. All experiments were replicated with distinct biological samples at least three times. Data were only excluded when samples are damaged or the time-lapses showed significant drifts during imaging.

## Supplementary information

Supplementary Information

Description of Additional Supplementary Files

Supplementary Movie 1

Supplementary Movie 2

Supplementary Movie 3

Supplementary Movie 4

Supplementary Movie 5

Supplementary Software

Source Data

## Data Availability

The data supporting this article are available from the corresponding author upon reasonable request. The source data underlying Figs. 4i, 6q, 7a, Supplementary Figs. 2a, 6a, and 6b are provided as a Source Data file.

## Code Availability

Scripts used in this study are available for download as a Supplementary Software. Matlab 2014a was used to code the program.
