# Supplementary material for: Extracellular matrix stiffness cues junctional remodeling for 3D tissue elongation
Source: Nat Commun. 2019 Jul 26;10:3339. doi: 10.1038/s41467-019-10874-x (PMC6659696; doi:10.1038/s41467-019-10874-x)
Supplement: Supplementary file 1 — Supplementary Information [file 41467_2019_10874_MOESM1_ESM.pdf]

Supplementary Information

3D Tissue elongation via ECM stiffness-cued junctional remodeling

Chen et. al.

Supplementary Figure 1

a *control^{RNAi}; indy-GFP*

DNA membrane GFP F-actin

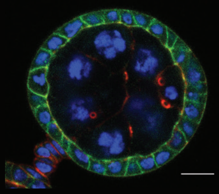
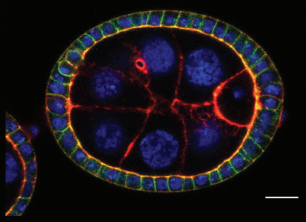

stage	**4**	**5**
number of cells	184 ± 34 s.d.	413 ± 50 s.d.
aspect ratio	1.17 ± 0.06 s.d.	1.34 ± 0.06 s.d.
volume (μm^3)	64,252 ± 1,3183 s.d.	107,630 ± 2,1727 s.d.
nurse cell nuclear diameter (μm)	N/A (polytene)	12.95 ± 1.22 s.d.

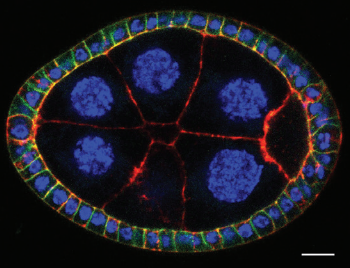
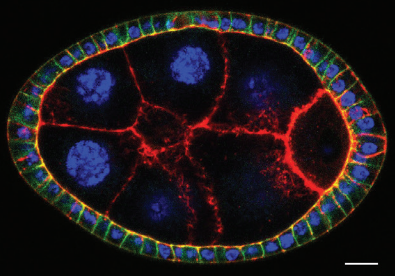

stage	**6A**	**6B**
number of cells	550 ± 44 s.d.	751 ± 66 s.d.
aspect ratio	1.42 ± 0.12 s.d.	1.54 ± 0.12 s.d.
volume (μm^3)	131,636 ± 32,104 s.d.	219,000 ± 8,0991 s.d.
nurse cell nuclear diameter (μm)	14.28 ± 1.95 s.d.	15.59 ± 2.61 s.d.

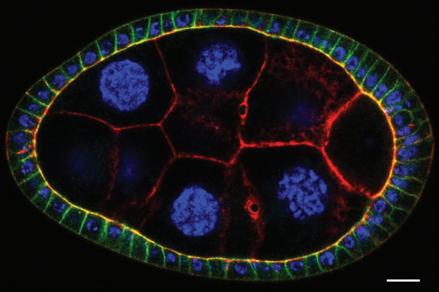
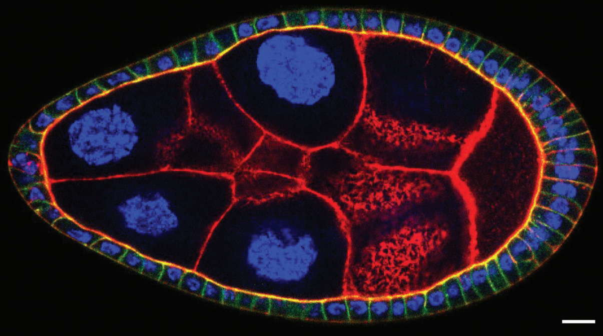

stage	**7**	**8**
number of cells	887 ± 33 s.d.	862 ± 28 s.d.
aspect ratio	1.82 ± 0.09 s.d.	1.91 ± 0.07 s.d.
volume (μm^3)	334062 ± 49880 s.d.	552705 ± 91295 s.d.
nurse cell nuclear diameter (μm)	19.86 ± 3.05 s.d.	23.64 ± 1.66 s.d.

10 μm

b

Stage	Duration by Lin&Spradling (h)	Duration by David&Merle (h)	Approx. range of follicle cell number	Approx. range of nurse cell nucleus diameter (μm)
4	6	9.16	125-350	N/A (polytene)
5	5	2.61	350-500	11-14
6A	3	8.45	500-650	14-15
6B			650-850	15-17
7	6	8.69	850+	17-21
8	6	5.21	850+	21+

Supplementary Figure 1| Representative wildtype egg chamber images and morphometrics.

a, Morphometric data for follicles staged by cell number, which generally correlate with classic morphological criteria. Values shown are average ± standard deviation (s.d.) **b**, Table showing durations of stages according to Lin and Spradling (1993) deduced from single ovariole transplants into *ovoD1* female hosts, or David and Merle (1968) deduced from representation of follicle stages in wildtype hosts; Approximate range of follicle cell number and nurse cell nucleus diameter for suggested staging. scale bars, 10 μm.

Supplementary Figure 2

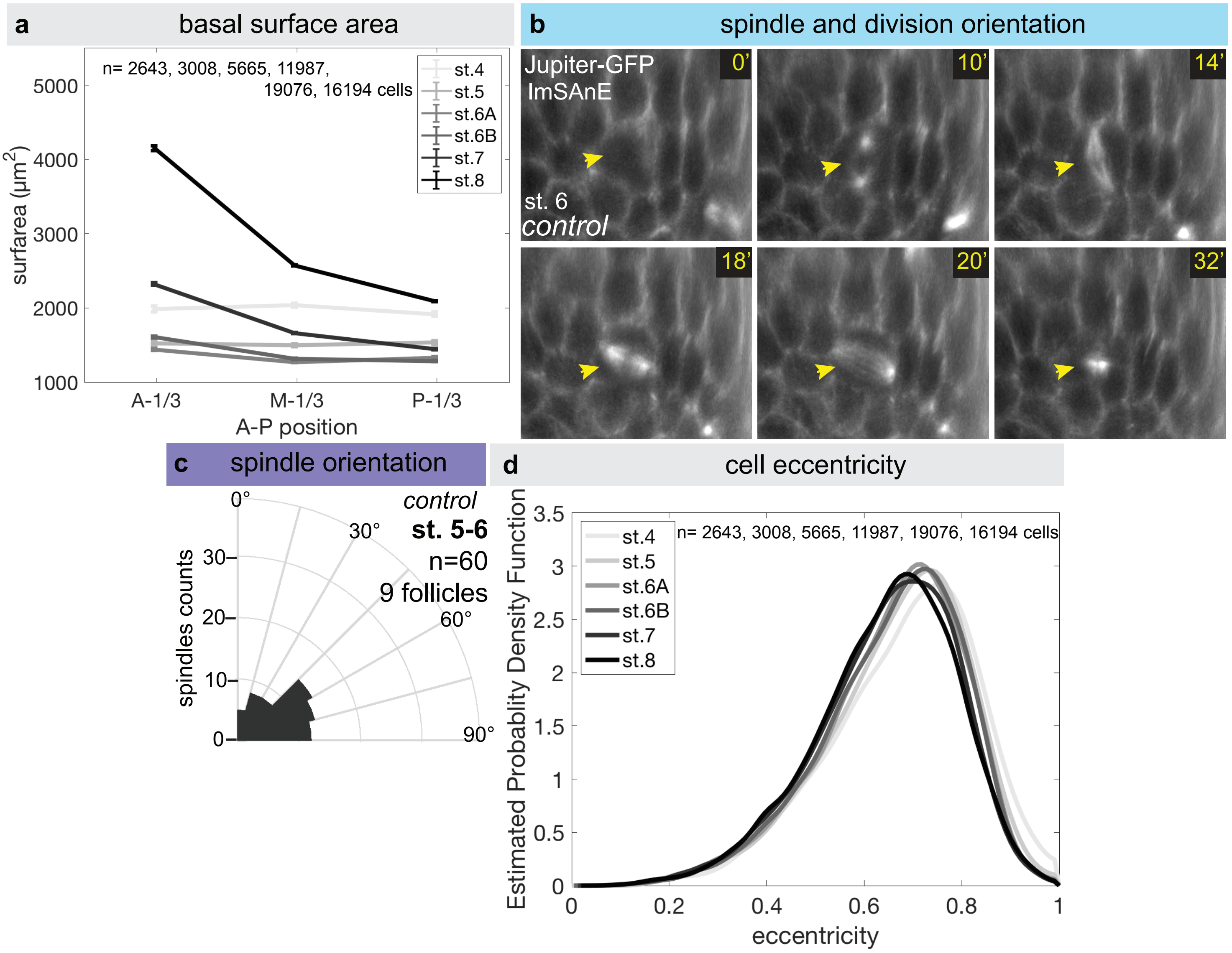

Supplementary Figure 2| Additional morphometrics and mitotic spindle orientation.

a, Mean basal surface area in wild type from stage 4-8 along the A-P meridian. (A-1/3: anterior one third; M-1/3: middle one third; P-1/3: posterior one third). **b,** Example from ImSAnE cylinder projection showing mitotic spindle (yellow arrowhead) in control stage 6 follicle rotating from initial latitudinal orientation to final AP-oriented cell division (see also **Supplementary Movie 3**). **c**, Quantitation of mitotic spindle orientations in wild type stage 5-6 follicles (0 º = latitudinal; 90 º = meridional). **d**, Distribution of cell eccentricities in wild type from stage 4-8. n, biologically independent samples. Error bars, s.e.m. Source data are provided as a Source Data file.

Supplementary Figure 3

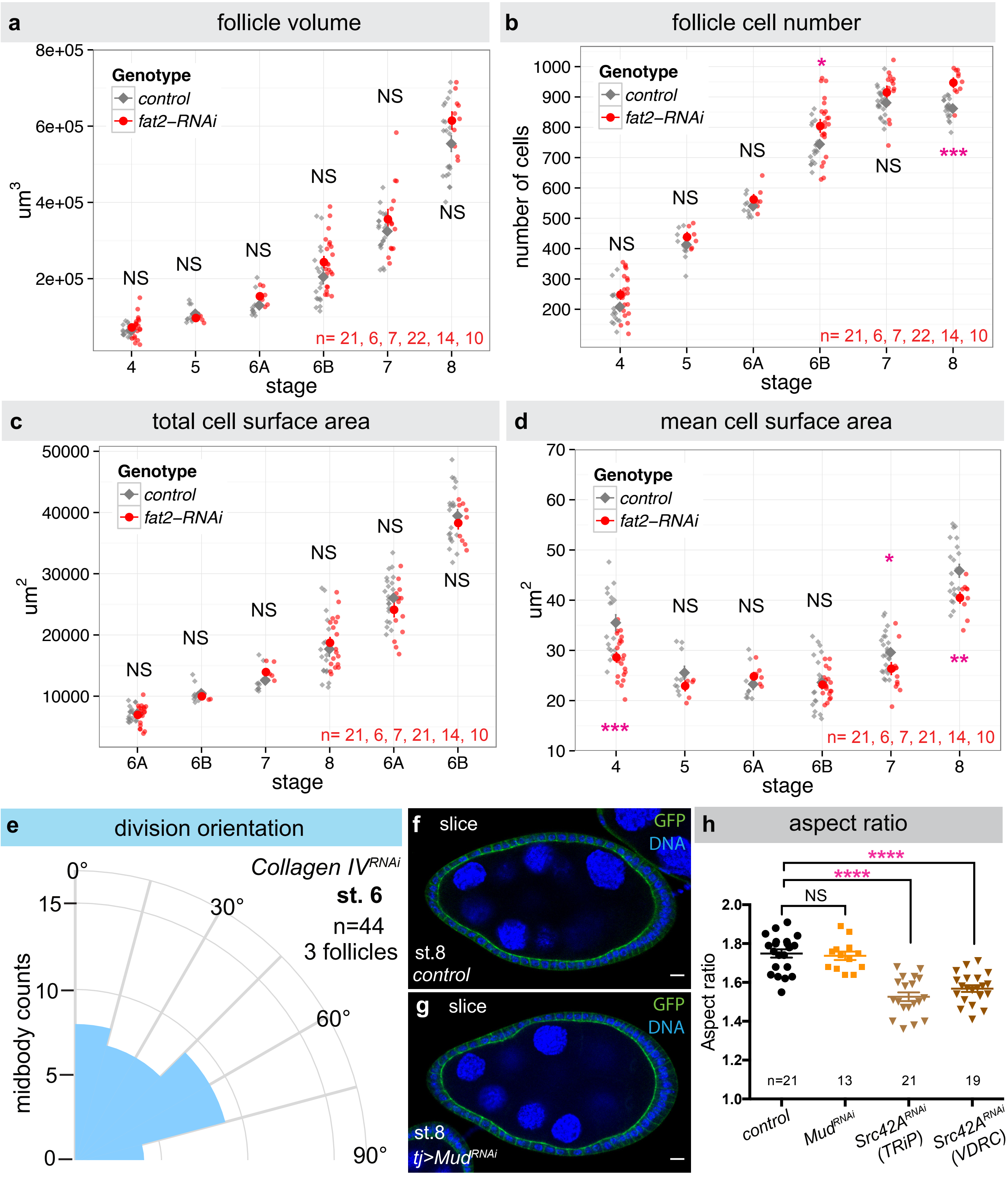

Supplementary Figure 3| Additional morphometric analyses of *fat2, Collagen IV, Mud, and Src42A*-depleted follicles.

a-d, Further morphometric data comparing control to *fat2*-depleted follicles with volume (**a**), P-values = 0.28, 0.19, 0.10, 0.27, 0.06; two-sided Welch's t-test, cell number (**b**), P-values = 0.05, 0.26, 0.25, 0.024, 0.15, 2.0e-04; two-sided Welch's t-test, total cell surface area (**c**), P-values = 0.73, 0.38, 0.11, 0.47, 0.17, 0.41; two-sided Welch's t-test, and mean cell surface area (**d**), P-values =9.1e-04, 0.13, 0.26, 0.77, 0.03, 0.003; two-sided Welch's t-test. **e**, Cell divisions remain ~randomly oriented at stage 6 in *Collagen IV*-depleted follicles. **f-g**, *Mud*-depleted follicles are able to elongate. **h,** Quantitation of follicle elongation in *Mud*-depleted and *Src42A*-depleted follicles stage 8 follicles. P-values = 0.69, <0.0001, <0.0001; two-sided Welch's t-test. n, biologically independent samples. NS, not significant, *P<0.5, **P<0.01, ***P<0.001, ****P<0.0001. scale bars, 10 µm. Error bars, s.e.m.

Supplementary Figure 4

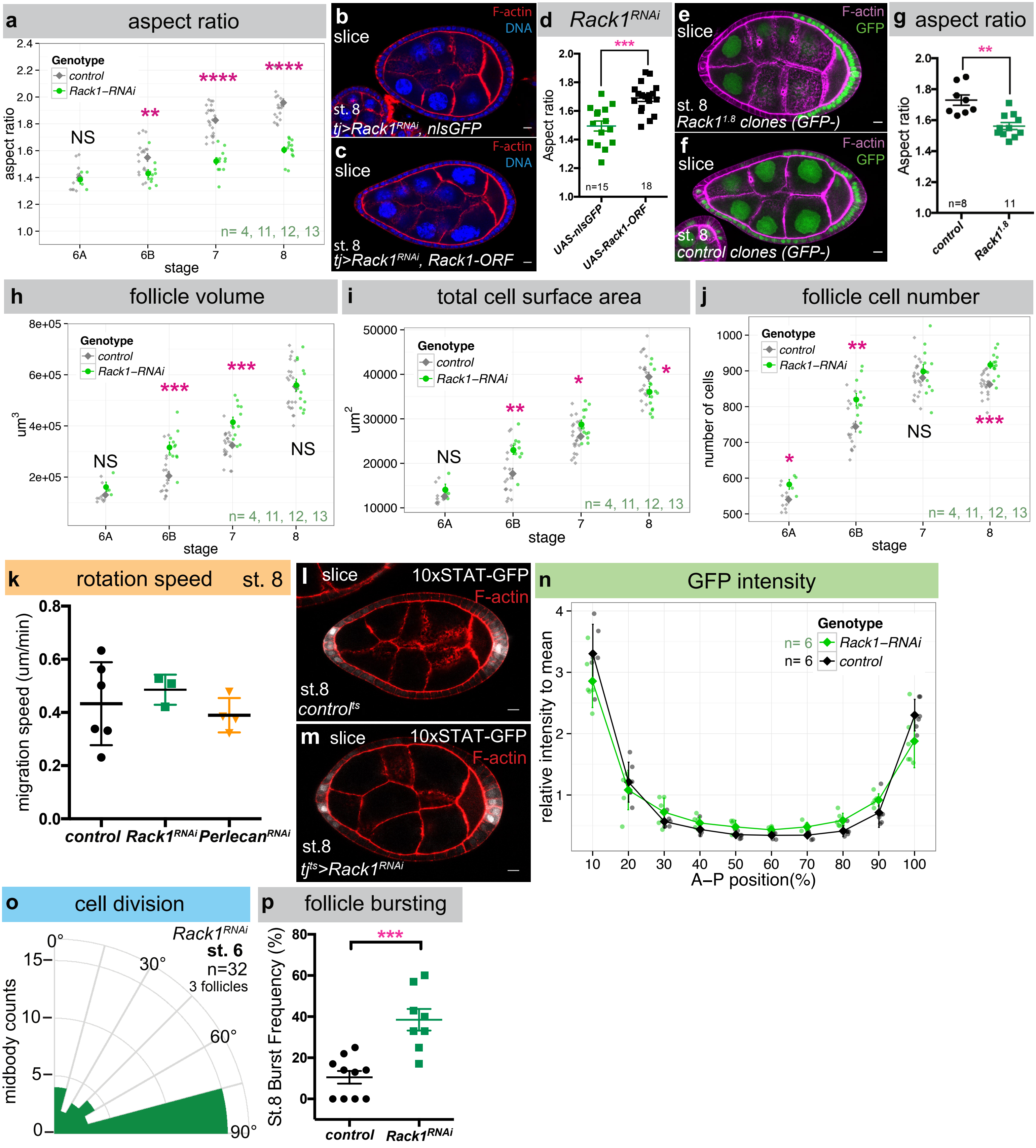

Supplementary Figure 4| Morphometrics, follicle rotation speed, STAT activity, cell division orientation, and follicle bursting of *Rack1*-depleted follicle.

a, Elongation of *Rack1*-depleted follicles diverges from control at stage 6B. **b-d,** *Rack1*-depleted round follicle phenotype is rescued by overexpression of the *Rack1*-ORF. P-value = 0.0001; two-sided Welch's t-test. **e-g**, Mitotic clones of a *Rack1* null allele (marked by absence of GFP) perturb elongation. P-value = 0.0013; two-sided Welch's t-test. **h-j**, Further morphometric data comparing control to *Rack1*-depleted follicles follicle with volume (**h**); P-values = 0.21, 5.2e-04, 8.5e-04, 0.86; two-sided Welch's t-test, total cell surface area (**i**); P-values = 0.34, 0.0019, 0.04, 0.04; two-sided Welch's t-test, and follicle cell number (**j**); P-values = 0.04, 0.003, 0.42, 0.0001; two-sided Welch's t-test. **k**, Both *Rack1 and Perlecan*-depleted follicles undergo rotation (see also **Supplementary Movie 4**). **l-n**, *Rack1*-depleted follicles show normal STAT activity gradients (grey) at poles. **o,** *Rack1*-depleted follicles undergo oriented cell divisions at stage 6 (n=32) similar to control. **p,** Bursting frequency of *Rack1*-depleted follicles under osmotic stress is increased compared to control follicles. n, biologically independent samples. NS, not significant, *P<0.5, **P<0.01, ***P<0.001, ****P<0.0001. scale bars, 10 µm. except for **n**, Error bars, s.e.m.

Supplementary Figure 5

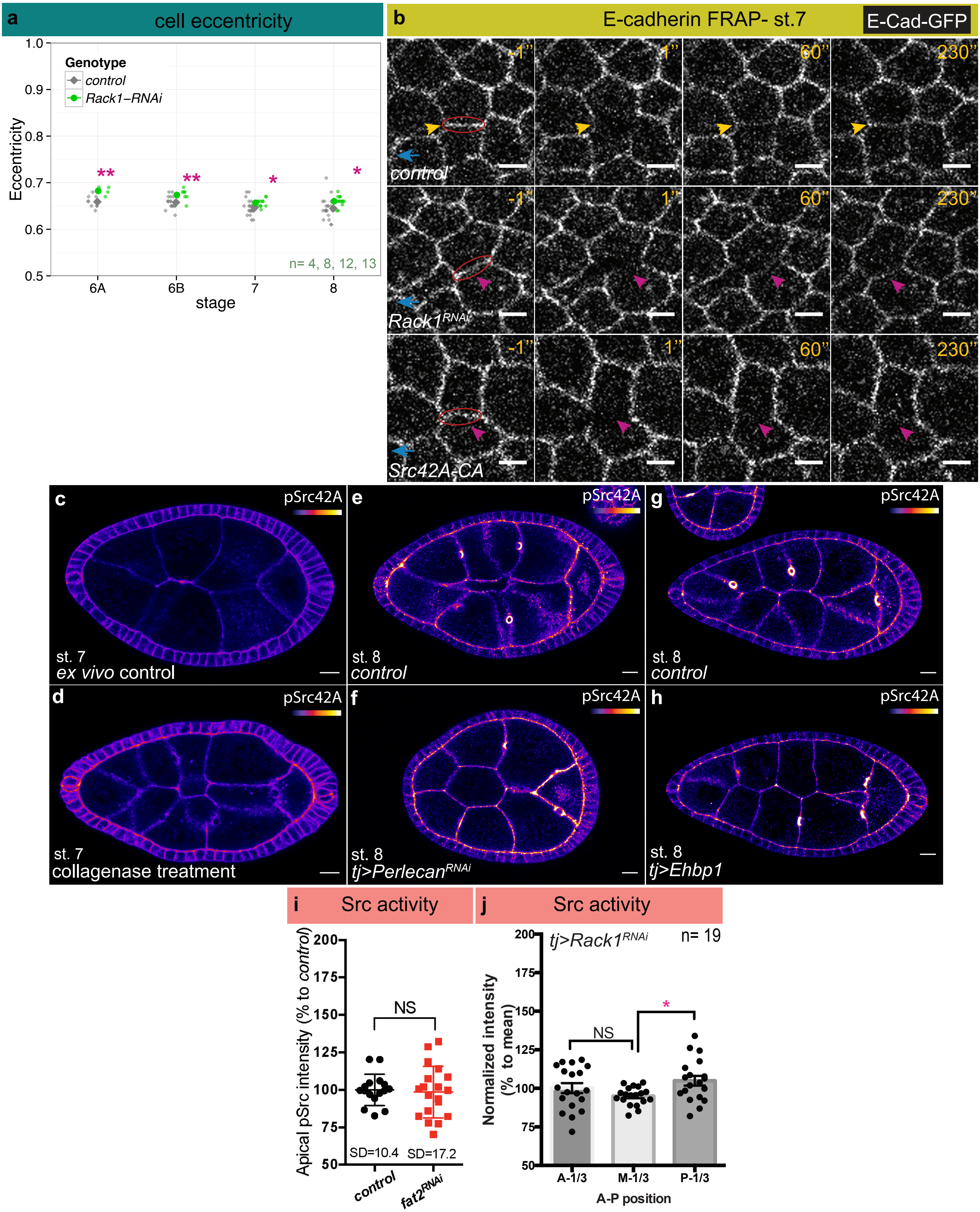

Supplementary Figure 5| Additional morphometrics of *Rack1*-depleted follicles, representative FRAP images, and representative images for Src activity measurements.
a, Cell eccentricity is slightly increased when *Rack1* is depleted. P-values = 0.0063, 0.0079, 0.017, and 0013; two-sided Welch's t-test. **b**, FRAP recovery is not through lateral diffusion. Yellow and magenta arrows mark photobleached junctions; blue arrows indicate direction of follicle rotation. (See also **Supplementary Movie 5**). **c-h**, Representative images from pSrc measurements in Figure 7 on **c-d**, *ex-vivo* culture control and collagenase treatment, **e-f**, control and *Perlecan-RNAi*, **g-h,** control and *UAS-Ehbp1*. **i**, *fat2*-depleted follicles show higher variation of phosphorylated Src at apical junctions compared to wild type. Error bars, standard deviation (SD), n= 16 and 19, P-value s = 0.76; two-sided Welch's t-test. **j**, Quantification of pSrc intensity in stage 8 *Rack1* depleted follicles along the A-P meridian (A-1/3: anterior one-third; M-1/3: middle-one third; P-1/3: posterior one-third). pSrc intensities were normalized to mean pSrc levels of each follicle. Error bars, s.e.m. P-value s =0.194 and 0.013; Two-sided paired sample t-test. n, biologically independent samples. Scale bars in **b**, 3 µm. Scale bars in **c-h**, 10 µm. NS, not significant, *P<0.5, **P<0.01.

Supplementary Figure 6

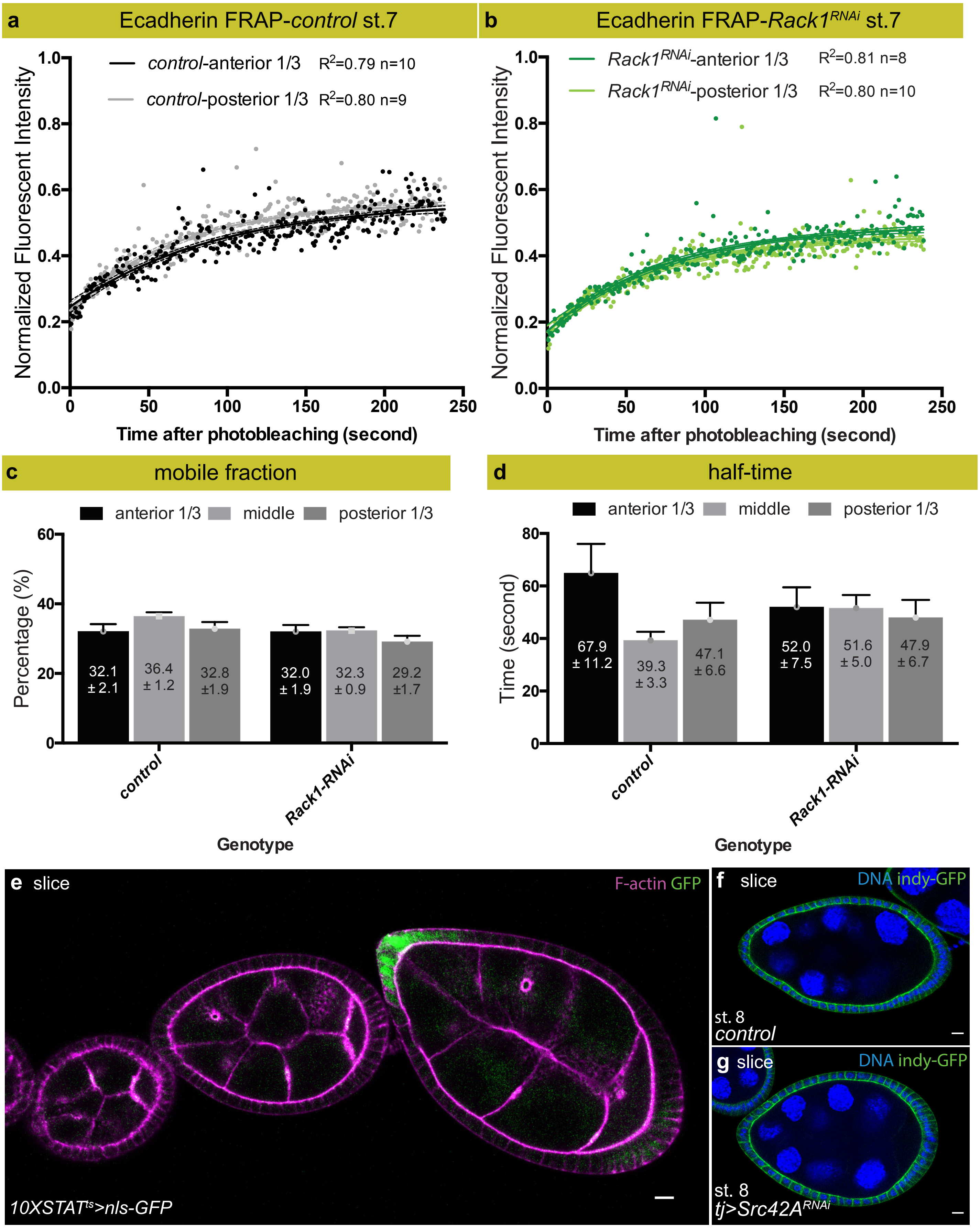

Supplementary Figure 6| Positional FRAP analyses, 10XSTAT-GAL4 expression pattern, and Src42A-RNAi phenotype.

a-b, FRAP analysis of Ecad-GFP in *control* and *Rack1*-depleted follicles at stage 7, performed at the anterior and posterior one-third. Dots represent mean of normalized intensity after photobleaching and lines are fitted curves with error bars showing 95% confidence intervals. **c**, Mobile fraction of Ecad-GFP is similar along the A-P meridian and in both genotypes. Error bars, 95% confidence intervals. **d**, Recovery half-time is increased at the anterior in *control* compared to the middle (equator); half-time at anterior, middle, and the posterior are similar when *Rack1* is depleted. Error bars show 95% confidence intervals. **e,** *10XSTAT-GAL4* drives transgene expression predominantly in follicle anterior at stage 7-8. **f-g,** *Src42A*-depleted follicles show impaired elongation. n, Sample size. Scale bars, 10 μm. Source data are provided as a Source Data file.

Supplemenary Table 1: Detailed Drosophila genotypes		
**Figure**	**Panel**	**Genotype**
**1**	**a-g**	*tj-GAL4/+; mcherry-RNAi/indy-GFP*
**2**	**a-e**	*tj-GAL4/+; mcherry-RNAi/indy-GFP*
**3**	**b-d**	*tj-GAL4/+; mcherry-RNAi/Jupiter-GFP*
	**f-i**	*tj-GAL4/+; mcherry-RNAi/indy-GFP*
**4**	**b-d**	*tj-GAL4/+; mcherry-RNAi/indy-GFP*
	**f-i**	*tj-GAL4/+; mcherry-RNAi/indy-GFP*
**5**	**a**	*tj-GAL4/+; mcherry-RNAi/indy-GFP*
	**b**	*tj-GAL4/fat2-RNAi; indy-GFP/+*
	**c**	*tj-GAL4/+; mcherry-RNAi/indy-GFP*<br>*tj-GAL4/fat2-RNAi; indy-GFP/+*
	**d**	*tj-GAL4/fat2-RNAi; indy-GFP/+*
	**e**	*tj-GAL4, tubP-GAL80[ts]/+; mcherry-RNAi/+*
	**f**	*tj-GAL4, tubP-GAL80[ts]/+; UAS-fzr/+*
	**g-h**	*tj-GAL4, tubP-GAL80[ts]/+; mcherry-RNAi/+*<br>*tj-GAL4, tubP-GAL80[ts]/+; mcherry-RNAi/indyGFP*<br>*tj-GAL4, tubP-GAL80[ts]/+; UAS-fzr/+*<br>*tj-GAL4, tubP-GAL80[ts]/+; UAS-fzr/indyGFP*
	**i-j**	*tj-GAL4/+; mcherry-RNAi/indy-GFP*<br>*tj-GAL4/fat2-RNAi; indy-GFP/+*
	**k-l**	*tj-GAL4/fat2-RNAi; indy-GFP/+*
	**m-o**	*tj-GAL4/+; mcherry-RNAi/indy-GFP*<br>*tj-GAL4/fat2-RNAi; indy-GFP/+*
**6**	**a**	*tj-GAL4/+; mcherry-RNAi/indy-GFP*
	**b**	*tj-GAL4/+; Rack1-RNAi/indy-GFP*
	**c**	*tj-GAL4, E-cadGFP[ki]/+; mcherry-RNAi/+*
	**d**	*tj-GAL4, E-cadGFP[ki]/+; Rack1-RNAi/+*
	**e**	*tj-GAL4/+; mcherry-RNAi/indy-GFP*<br>*tj-GAL4/+; Rack1-RNAi/indy-GFP*
	**f**	*tj-GAL4/+; mcherry-RNAi/indy-GFP*
	**g**	*tj-GAL4/+; Rack1-RNAi/indy-GFP*
	**h**	*tj-GAL4/+; mcherry-RNAi/indy-GFP*<br>*tj-GAL4, E-cadGFP[ki]/+; mcherry-RNAi/+*<br>*tj-GAL4/+; Rack1-RNAi/indy-GFP*<br>*tj-GAL4, E-cadGFP[ki]/+; Rack1-RNAi/+*
	**i**	*tj-GAL4/+; mcherry-RNAi/indy-GFP*
	**j**	*tj-GAL4/+; Rack1-RNAi/indy-GFP*
	**k**	*tj-GAL4/+; mcherry-RNAi/indy-GFP*<br>*tj-GAL4/+; Rack1-RNAi/indy-GFP*
	**l**	*tj-GAL4/+; mcherry-RNAi/indy-GFP*<br>*tj-GAL4/+; UAS-Src42A-CA/indy-GFP*
	**m**	*w1118; tj-GAL4, tubP-GAL80[ts]/+; Rack1-RNAi/+*<br>*tj-GAL4, tubP-GAL80[ts]/Src42A[26-1]; Rack1-RNAi/+*
	**n-o**	*tj-GAL4/+; Rack1-RNAi/indy-GFP*
	**p-s**	*tj-GAL4/+; mcherry-RNAi/indy-GFP*<br>*tj-GAL4/+; Rack1-RNAi/indy-GFP*

7	a-c	*tj-GAL4, E-cadGFP[ki]/+; mcherry-RNAi/+*
		tj-GAL4, E-cadGFP[ki]/+; Rack1-RNAi/+
		tj-GAL4, E-cadGFP[ki]/+; UAS-Src42A-CA/+
		tj-GAL4, vkg-GFP/+; mcherry-RNAi/+
	d	*yw; tj-GAL4/+; Dr/+*
		yw; tj-GAL4/+; UAS-mcherry-Ehbp1/Dr
		tj-GAL4/+; mcherry-RNAi/indy-GFP
	e-f	*tj-GAL4/+; Perlecan(trol)-RNAi/indy-GFP*
		tj-GAL4/+; mcherry-RNAi/indy-GFP
		tj-GAL4/+; Perlecan(trol)-RNAi/indy-GFP
	g	*tj-GAL4/+; mcherry-RNAi/indy-GFP*
		tj-GAL4/fat2-RNAi; indy-GFP/+
		tj-GAL4/+; Perlecan(trol)-RNAi/indy-GFP
	h-i	*tj-GAL4/+; mcherry-RNAi/indy-GFP*
	j	*w; 10xSTAT-GAL4/+; UAS-nls-GFP/tubP-GAL80[ts]*
	k	*w; 10xSTAT-GAL4/+; Rack1-RNAi/tubP-GAL80[ts]*
	l	*w; 10xSTAT-GAL4/+; mcherry-RNAi/tubP-GAL80[ts]*
		w; 10xSTAT-GAL4/+; Rack1-RNAi/tubP-GAL80[ts]
		w; 10xSTAT-GAL4/+; UAS-Src42A-CA/tubP-GAL80[ts]
S1	a-b	*tj-GAL4/+; mcherry-RNAi/indy-GFP*
S2	a	*tj-GAL4/+; mcherry-RNAi/indy-GFP*
	b-c	*tj-GAL4/+; mcherry-RNAi/Jupiter-GFP*
	d	*tj-GAL4/+; mcherry-RNAi/indy-GFP*
S3	a-d	*tj-GAL4/+; mcherry-RNAi/indy-GFP*
		tj-GAL4/fat2-RNAi; indy-GFP/+
	e	*tj-GAL4/vkg-RNAi; cg25c-RNAi/Jupiter-GFP*
	f	*tj-GAL4/+; mcherry-RNAi/indy-GFP*
	g	*tj-GAL4/+; Mud-RNAi/indy-GFP*
	h	*tj-GAL4/+; mcherry-RNAi/indy-GFP*
		tj-GAL4/+; Mud-RNAi/indy-GFP
		tj-GAL4/+; Src42A-RNAi(TRiP)/indy-GFP
		tj-GAL4/Src42A-RNAi(VDRC-KK)/; indy-GFP/+
	a	*tj-GAL4/+; mcherry-RNAi/indy-GFP*
		tj-GAL4/+; Rack1-RNAi/indy-GFP
	b	*tj-GAL4, tubP-GAL80[ts]/+; Rack1-RNAi/UAS-nlsGFP*
	c	*tj-GAL4, tubP-GAL80[ts]/+; Rack1-RNAi/UAS-Rack1-ORF-3xHA*
	d	*tj-GAL4, tubP-GAL80[ts]/+; Rack1-RNAi/UAS-nlsGFP*
		tj-GAL4, tubP-GAL80[ts]/+; Rack1-RNAi/UAS-Rack1-ORF-3xHA

S4	e	ubi-eGFP-FRT40/Rack1[1.8]-FRT40A; GR1-GAL4, UAS-FLP/+
	f	ubi-eGFP-FRT40/FRT40A; GR1-GAL4, UAS-FLP/+
	g	ubi-eGFP-FRT40/Rack1[1.8]-FRT40A; GR1-GAL4, UAS-FLP/+
		ubi-eGFP-FRT40/FRT40A; GR1-GAL4, UAS-FLP/+
	h-j	*tj-GAL4/+; mcherry-RNAi/indy-GFP*
		tj-GAL4/+; Rack1-RNAi/indy-GFP
	k	*tj-GAL4/+; mcherry-RNAi/indy-GFP*
		tj-GAL4/+; Rack1-RNAi/indy-GFP
		tj-GAL4/+; Perlecan(trol)-RNAi/indy-GFP
	l	*tj-GAL4, tubP-GAL80[ts]/+; 10XSTAT-GFP/mcherry-RNAi*
	m	*tj-GAL4, tubP-GAL80[ts]/+; 10XSTAT-GFP/Rack1-RNAi*
	n	*tj-GAL4, tubP-GAL80[ts]/+; 10XSTAT-GFP/mcherry-RNAi*
		tj-GAL4, tubP-GAL80[ts]/+; 10XSTAT-GFP/Rack1-RNAi
S5	a	*tj-GAL4/+; mcherry-RNAi/indy-GFP*
		tj-GAL4/+; Rack1-RNAi/indy-GFP
	b	*tj-GAL4, E-cadGFP[ki]/+; mcherry-RNAi/+*
		tj-GAL4, E-cadGFP[ki]/+; Rack1-RNAi/+
		tj-GAL4, E-cadGFP[ki]/+; UAS-Src42A-CA/+
	c-d	*tj-GAL4, vkg-GFP/+; mcherry-RNAi/+*
	e	*tj-GAL4/+; mcherry-RNAi/indy-GFP*
	f	*tj-GAL4/+; Perlecan(trol)-RNAi/indy-GFP*
	g	yw; tj-GAL4/+; Dr/+
	h	yw; tj-GAL4/+; UAS-mcherry-Ehbp1/Dr
	i	*tj-GAL4/+; mcherry-RNAi/indy-GFP*
		tj-GAL4/fat2-RNAi; indy-GFP/+
	j	*tj-GAL4/+; Rack1-RNAi/indy-GFP*
S6	a-d	*tj-GAL4, E-cadGFP[ki]/+; mcherry-RNAi/+*
		tj-GAL4, E-cadGFP[ki]/+; Rack1-RNAi/+
	e	*w; 10xSTAT-GAL4/+; UAS-nls-GFP/tubP-GAL80[ts]*
	f	*tj-GAL4/+; mcherry-RNAi/indy-GFP*
	g	*tj-GAL4/+; Src42A-RNAi(TRiP)/indy-GFP*

Supplementary Table 2 : Antibodies and reagents			
**Antibody/Reagent**	**Dilution used**	**Provider**	**Catalogue Number**
Mouse monoclonal Anti-GFP (3E6)	1:50	ThermoFisher	A11120
Goat anti-Mouse IgG (H+L) Cross-Adsorbed Secondary Antibody, Alexa Fluor 488	1:300	ThermoFisher	A11001
Goat anti-Rabbit IgG (H+L) Cross-Adsorbed Secondary Antibody, Alexa Fluor 488	1:300	ThermoFisher	A11008
Goat anti-Rabbit IgG (H+L) Cross-Adsorbed Secondary Antibody, Alexa Fluor 647	1:300	ThermoFisher	A21244
Donkey anti-Mouse IgG (H+L) Highly Cross-Adsorbed Secondary Antibody, Alexa Fluor 647	1:300	ThermoFisher	A31571
Phospho-Src Family (Tyr416)	1:500	ThermoFisher	2101
Phospho-Src42A (Tyr400)	1:500	Shigeo Hayashi lab (Shindo, M., et al. 2008)	N/A
FasIII	1:100	Developmental Studies Hybridoma Bank	7G10
Src42A	1:200	Tetsuya Kojima lab (Takahashi, M., et. al. 2005)	N/A
Schneider’s Drosophila medium(1x)	N/A	gibco	21720-024
Fetal bovine serum defined	N/A	Hyclone	SH30070.03
insulin	N/A	sigma	I5500
Penicillin-Streptomycin (10,000 U/mL)	N/A	gibco	15140
Phalloidin-TRITC	N/A	Sigma	P1951
Alexa Fluor 647 Phalloidin	N/A	ThermoFisher	A22287
DAPI	N/A	Sigma	D9542
Bovine Serum Albumin	N/A	Sigma	A3912

Supplementary Methods

Complete sequence of the five tandem copies of the Socs36E enhancer fragment for creating *10XSTAT-GAL4* is below:

GCGGCCGCGAACCACTCAGAGTGCCTGCGTGTGTGTGTGTGTGTGCGTGTGAGTCCG
CTTGTGTATCTGTGAGAGTGAGTGCGAGTACGAGTATCTTTGTATCTGCTGGTTCGCCT
GGGCTGTCTGTCACTTCTCAGAAATCGGTCTAAATTAGAAAATGCGTTTCTGGGAATTT
CGTATCGCTCTTTTGGGCCCACGGAGCTCAGCGAACAACATATATAAAAAAATATACAT
ATATATGTATATTGTCAGTATATATTGTAGCCATTCCCCTTACGTCCAGTTCGCGGAATA
TTGCGCCTTTCCCTAATAAATTTATCAATGGCATTTGCAAATATTATATAGCAAACAGGG
CAACTATTTATTTTGCTTAGTAATTTATGCAAACAGCGAGCGGTTCAGGGAATTCCAATT
ACCACAATGTGGTAAACACCTAAGACAGTTTTGTATACCGGTGAACCACTCAGAGTGCC
TGCGTGTGTGTGTGTGTGTGCGTGTGAGTCCGCTTGTGTATCTGTGAGAGTGAGTGCG
AGTACGAGTATCTTTGTATCTGCTGGTTCGCCTGGGCTGTCTGTCACTTCTCAGAAATC
GGTCTAAATTAGAAAATGCGTTTCTGGGAATTTCGTATCGCTCTTTTGGGCCCACGGAG
CTCAGCGAACAACATATATAAAAAAATATACATATATATGTATATTGTCAGTATATATTGT
AGCCATTCCCCTTACGTCCAGTTCGCGGAATATTGCGCCTTTCCCTAATAAATTTATCAA
TGGCATTTGCAAATATTATATAGCAAACAGGGCAACTATTTATTTTGCTTAGTAATTTATG
CAAACAGCGAGCGGTTCAGGGAATTCCAATTACCACAATGTGGTAAACACCTAAGACA
GTTTTGTATAAGCTTGAACCACTCAGAGTGCCTGCGTGTGTGTGTGTGTGTGCGTGTGA
GTCCGCTTGTGTATCTGTGAGAGTGAGTGCGAGTACGAGTATCTTTGTATCTGCTGGTT
CGCCTGGGCTGTCTGTCACTTCTCAGAAATCGGTCTAAATTAGAAAATGCGTTTCTGGG
AATTTCGTATCGCTCTTTTGGGCCCACGGAGCTCAGCGAACAACATATATAAAAAAATAT
ACATATATATGTATATTGTCAGTATATATTGTAGCCATTCCCCTTACGTCCAGTTCGCGG
AATATTGCGCCTTTCCCTAATAAATTTATCAATGGCATTTGCAAATATTATATAGCAAACA
GGGCAACTATTTATTTTGCTTAGTAATTTATGCAAACAGCGAGCGGTTCAGGGAATTCC
AATTACCACAATGTGGTAAACACCTAAGACAGTTTTGTATAAGCTTGAACCACTCAGAGT
GCCTGCGTGTGTGTGTGTGTGTGCGTGTGAGTCCGCTTGTGTATCTGTGAGAGTGAGT
GCGAGTACGAGTATCTTTGTATCTGCTGGTTCGCCTGGGCTGTCTGTCACTTCTCAGAA
ATCGGTCTAAATTAGAAAATGCGTTTCTGGGAATTTCGTATCGCTCTTTTGGGCCCACG
GAGCTCAGCGAACAACATATATAAAAAAATATACATATATATGTATATTGTCAGTATATAT
TGTAGCCATTCCCCTTACGTCCAGTTCGCGGAATATTGCGCCTTTCCCTAATAAATTTAT
CAATGGCATTTGCAAATATTATATAGCAAACAGGGCAACTATTTATTTTGCTTAGTAATTT
ATGCAAACAGCGAGCGGTTCAGGGAATTCCAATTACCACAATGTGGTAAACACCTAAGA
CAGTTTTGTATGCATGCGAACCACTCAGAGTGCCTGCGTGTGTGTGTGTGTGTGCGTG
TGAGTCCGCTTGTGTATCTGTGAGAGTGAGTGCGAGTACGAGTATCTTTGTATCTGCTG
GTTCGCCTGGGCTGTCTGTCACTTCTCAGAAATCGGTCTAAATTAGAAAATGCGTTTCT
GGGAATTTCGTATCGCTCTTTTGGGCCCACGGAGCTCAGCGAACAACATATATAAAAAA
ATATACATATATATGTATATTGTCAGTATATATTGTAGCCATTCCCCTTACGTCCAGTTCG
CGGAATATTGCGCCTTTCCCTAATAAATTTATCAATGGCATTTGCAAATATTATATAGCA
AACAGGGCAACTATTTATTTTGCTTAGTAATTTATGCAAACAGCGAGCGGTTCAGGGAA
TTCCAATTACCACAATGTGGTAAACACCTAAGACAGTTTTGTATGCGGCCGC
