# Supplementary material for: Extracellular matrix stiffness cues junctional remodeling for 3D tissue elongation
Source: Nat Commun. 2019 Jul 26;10:3339. doi: 10.1038/s41467-019-10874-x (PMC6659696; doi:10.1038/s41467-019-10874-x)
Supplement: Supplementary file 2 — Description of Additional Supplementary Files [file 41467_2019_10874_MOESM2_ESM.pdf]

Description of Additional Supplementary Files

File Name: Supplementary Movie 1
Description: Sample 3D reconstruction of a segmented stage 8 follicle from the morphometric workflow. Cells are colour-coded for topology as in Fig. 1g.

File Name: Supplementary Movie 2
Description: ImSAnE cylinder projection of live-imaged follicle expressing Jupiter-GFP (gray) to visualize mitotic spindles and midbodies.

File Name: Supplementary Movie 3
Description: Time-lapse video showing mitotic spindle (grey) in control stage 6 follicle rotates from latitudinal orientation to final A-P-oriented cell division as in Supplementary Figure 2b.

File Name: Supplementary Movie 4
Description: Rack1-depleted follicle is not defective in rotation. Time-lapse video showing a stage 8 Rack1-depleted follicle marked with histone (magenta) and follicle cell membrane (green) rotated ex vivo. Scale bar, 10 µm.

File Name: Supplementary Movie 5
Description: Time-lapse videos showing FRAP in stage 7 control (left panel), Rack1- depleted follicle (middle panel), and Src42A-hyperactivated follicle (right panel) as in Fig. 7a-c. One latitudinally-oriented junction marked with E-cadherin-GFP(gray) of each genotype was bleached during 8-9 seconds, followed by recovery. Scale bars, 3 µm.
